# Stellate cell computational modeling predicts signal filtering in the molecular layer circuit of cerebellum

**DOI:** 10.1038/s41598-021-83209-w

**Published:** 2021-02-16

**Authors:** Martina Francesca Rizza, Francesca Locatelli, Stefano Masoli, Diana Sánchez-Ponce, Alberto Muñoz, Francesca Prestori, Egidio D’Angelo

**Affiliations:** 1 Department of Brain and Behavioral Sciences, University of Pavia, Via Forlanini 6, 27100 Pavia, Italy; 2 Brain Connectivity Center, IRCCS Mondino Foundation, Pavia, Italy; 3 Centro de Tecnología Biomédica (CTB), Technical University of Madrid, Madrid, Spain; 4 Departamento de Biología Celular, Complutense University of Madrid, Madrid, Spain

**Keywords:** Computational biology and bioinformatics, Neuroscience, Physiology

## Abstract

The functional properties of cerebellar stellate cells and the way they regulate molecular layer activity are still unclear. We have measured stellate cells electroresponsiveness and their activation by parallel fiber bursts. Stellate cells showed intrinsic pacemaking, along with characteristic responses to depolarization and hyperpolarization, and showed a marked short-term facilitation during repetitive parallel fiber transmission. Spikes were emitted after a lag and only at high frequency, making stellate cells to operate as delay-high-pass filters. A detailed computational model summarizing these physiological properties allowed to explore different functional configurations of the parallel fiber—stellate cell—Purkinje cell circuit. Simulations showed that, following parallel fiber stimulation, Purkinje cells almost linearly increased their response with input frequency, but such an increase was inhibited by stellate cells, which leveled the Purkinje cell gain curve to its 4 Hz value. When reciprocal inhibitory connections between stellate cells were activated, the control of stellate cells over Purkinje cell discharge was maintained only at very high frequencies. These simulations thus predict a new role for stellate cells, which could endow the molecular layer with low-pass and band-pass filtering properties regulating Purkinje cell gain and, along with this, also burst delay and the burst-pause responses pattern.

## Introduction

The stellate cells (SCs) are inhibitory interneurons of the cerebellum molecular layer (ML) that were first identified in histological preparations by Golgi^1^ and Cajal^2,3^. Electrophysiological recordings in vivo^4,5^ revealed their inhibitory nature. Since then, several observations have been reported on SC morphology^6,7^ and molecular properties^8–21^, but the way SCs control cerebellar functioning remained elusive. One of the reasons is that isolating the contribution of SCs from that of other microcircuit elements (especially the basket cells) is experimentally impractical leaving several functional hypotheses undetermined. To face the issue, we have measured SCs electroresponsiveness and their activation by parallel fiber (PF) bursts with the aim of generating precise models, which had then been used to explore the computational impact of these neurons on Purkinje cells (PCs).

SCs populate the outer two thirds of the cerebellum ML and provide inhibition to PCs. Their dendrites are organized isotropically around the soma and lay on the parasagittal plane, where they are intercalated with the surrounding PC dendrites. The axon travels a short distance along the transverse plane, following the PFs, and terminates with inhibitory synapses on PC dendrites as well as on other SCs generating feed-forward inhibition^5,8–11^. Several studies have suggested that, in combination with the direct excitatory pathway provided by PF-PC synapses, feed-forward inhibition may assure effective motor performance^9,12–15^ and learning^16,22^. Inhibition provided by molecular layer interneurons (MLI) proved also able to regulate the bandwidth of PC responses in spots and beams^17,18^. Despite the importance of SCs, there is little knowledge on their mechanisms of action [for a recent review see^20^].

The electrophysiological properties of SCs have been identified only in part. SCs fire spontaneously in the 1–35 Hz range, both in vitro and in vivo^8,23,24^. The firing rate is dynamically regulated by several ionic channels, including T-type Ca^2+^ channels and A-type K^+^ channels^25,26^. Evidence has also been provided about the receptors and currents involved in synaptic transmission^27^. Given that a full characterization of all the relevant mechanisms is unavailable at the moment, a first hypothesis on stellate cell role could be generated through computational modeling, which can synthesize morpho-electrical information in a coherent rule-based framework (e.g. see the case of granule cell (GrC), Golgi cells and PCs, just to remain in the cerebellum)^28–35^.

Initial attempts at modeling ML functions involved simplified representations of MLI. Those models have shown the impact of increasing or decreasing SC transmission strength under the assumption of linear rate coding and in the absence of short-term plasticity^36,37^. However, it is now clear from several studies that detailed membrane and synaptic dynamics are critical to understand the way a neural circuit operates^38,39^. Therefore, we have recorded electroresponsive and synaptic dynamics of SC and used them to generate detailed computational models through well-defined workflows for model construction and validation^34,35,40^. The models, based on accurate morphologies and membrane mechanisms, faithfully reproduced the whole set of available experimental data. Importantly, a marked short-term facilitation at PF-SC synapses, as well as inhibitory transmission from other SCs, proved to be instrumental to modulate SC response curves to input bursts. The SC properties reverberated onto the PCs generating low-pass and band-pass filtering of PF signals^41^. These observations imply that SCs transform the ML in a filter that limits the response bandwidth of on-beam PCs into the low-frequency range, leading to new hypotheses on how the cerebellar cortex processes incoming signals.

## Methods

### Electrophysiological recordings

All experiments were conducted in accordance with European guidelines for the care and use of laboratory animals (Council Directive 2010/63/EU) and approved by the Ethical Committee of Italian Ministry of Health (628/2017-PR). We performed whole-cell patch-clamp recordings (WCR) from SCs in acute cerebellar slices from P18–P25 male and female C57/BL6 mice. Briefly, mice were killed by decapitation after deep anesthesia with halothane (Sigma-Aldrich). The cerebellum was gently removed, and the vermis was isolated and fixed on the stage of a vibroslicer with cyanoacrylic glue. Acute 220-μm-thick slices were cut in the coronal plane in a cold (2–3 °C) oxygenated bicarbonate-buffered saline solution (Kreb's solution) and maintained at room temperature for at least 1 h, before being transferred to a recording chamber. The slices were continuously perfused at a rate of 1.5 ml/min with oxygenated Kreb’s solution and maintained at 32 °C with a Peltier feedback device (TC-324B, Warner Instrument). The Kreb's solution contained the following (in mM): NaCl 120, KCl 2, MgSO_4_ 1.2, NaHCO_3_ 26, KH_2_PO_4_ 1.2, CaCl_2_ 2, glucose 11 (pH 7.4 when equilibrated with 95%O_2_–5%CO_2_). SR 95,531 (gabazine; 10 μM, Abcam) and strycnine (1 μM, Abcam) were added to the bath solution in order to block inhibitory synaptic inputs. Slices were visualized in an upright epifluorescence microscope (Axioskop 2 FS, Zeiss) equipped with a × 63, 0.9 NA water-immersion objective. Patch pipettes were fabricated from thick-walled borosilicate glass capillaries (Sutter Instruments) by means of a Sutter P-1000 horizontal puller (Sutter Instruments). Recordings were performed using a Multiclamp 700B [− 3 dB; cutoff frequency (fc), 10 kHz], sampled with Digidata 1550 interface, and analyzed off-line with pClamp10 software (Molecular Devices). SCs were recorded in the outer two-thirds of the molecular layer, where they are the only neuronal species present^6,42^.

PF stimulation was performed with a large-bore patch pipette filled with Kreb's solution and placed across the molecular layer ~ 200 µm from the recording electrode. The stimulus intensity ranged from 10 to 50 V with duration of 0.2 ms. In order to analyze short-term dynamics during repetitive stimulation, trains of 10–20 pulses @ 50, 100 and 200 Hz were applied.

All data are reported as mean ± SEM. Means were compared by a Student's *t*-test or by one-way parametric analysis of variance (ANOVA). Where appropriate, data were further assessed by conducting the Tukey post hoc test. The analysis was two-sided, with level of significance α = 0.05.

#### Whole-cell recording properties

The stability of WCR can be influenced by modification of series resistance (R_s_). To ensure that R_s_ remained stable during recordings, passive electrode-cell parameters were monitored throughout the experiments. In each recording, once in the whole-cell configuration, the current transients elicited by 10 mV hyperpolarizing pulses from the holding potential of − 70 mV in voltage-clamp mode showed a biexponential relaxation. Membrane capacitance (C_m_ = 6.7 ± 0.5 pF; n = 23) was measured from the capacitive charge (the area underlying current transients) and series resistance (R_s_ = 27.1 ± 3.1 MΩ; n = 23) was calculated as R_s_ = τ_VC_/C_m_. The input resistance (R_In_ = 1.3 ± 0.1 GΩ; n = 23) was computed from the steady-state current flowing after termination of the transient. The 3-dB cutoff frequency of the electrode-cell system, *f*_VC_ (1.2 ± 0.1; n = 23), was calculated as *f*_VC_ = (2π ∙ 2τ_VC_)^−1^. Series resistance was constantly monitored throughout the experiment. In analyzed recording periods, R_s_ was constant within 20%.

#### Stellate cell excitability

Pipette had a resistance of 7–10 MΩ before seal formation with a filling solution containing the following (in mM): potassium gluconate 126, NaCl 4, HEPES 5, glucose 15, MgSO_4_ · 7H_2_O 1, BAPTA-free 0.1, BAPTA-Ca^2+^ 0.05, Mg^2+^-ATP 3, Na^+^-GTP 0.1, pH adjusted to 7.2 with KOH. Just after obtaining the cell-attached configuration, electrode capacitance was carefully cancelled to allow for electronic compensation of pipette charging during subsequent current-clamp recordings. After switching to current clamp, intrinsic excitability was investigated by setting the holding current at 0 pA and injecting 2 s steps of current (from − 16 to 20 pA in a 4 pA increment). Action potential threshold (AP_thr_) was measured along the rising phase of membrane potential responses to step current injections. The AP_thr_ was identified at the flexus starting the regenerative process (a procedure that was improved by taking the second derivative of membrane potential). Action potential duration at half amplitude, half-width, (AP_HW_) was measured at the midpoint between the threshold and the peak. The amplitude of the action potential (AP_Ampl_) was estimated as the difference between the threshold and the maximum reached potential. The amplitude of the action potential afterhyperpolarization (AP_AHP_) was estimated as the difference between threshold and the lowest potential after the peak. Action potential frequency (AP_Freq_) was measured by dividing the number of spikes by step duration and spike times were used to calculate interspike intervals (ISI). Peri-stimulus time histograms (PSTHs) were constructed for the analysis of responses to 100 Hz-10 pulses stimulation. To optimize PSTH resolution, a 40 ms bin width was used.

#### Synaptic currents

Pipette had a resistance of 4–6 MΩ before seal formation with a filling solution containing the following (in mM): 81 Cs_2_SO_4_, 4 NaCl, 2 MgSO_4_, 1 QX-314 (lidocaine *N*-ethyl bromide), 0.1 BAPTA-free, 0.05 BAPTA-Ca^2+^, 15 glucose, 3 Mg^2+^-ATP, 0.1 Na^+^-GTP and 15 HEPES, pH adjusted to 7.2 with CsOH. SCs were voltage-clamped at -70 mV. The EPSC_S_ were elicited at three types of high-frequency trains (20 pulses @ 50, 100 and 200 Hz). Each train stimulation sweep was separated from the preceding one by at least 30 s. EPSC amplitude value was calculated as the difference between peak and base. The facilitation during the repetitive stimulation was computed, for each Δt, by normalizing the amplitudes to the first EPSC.

### Tissue preparation for morphological reconstructions

For morphological reconstruction of MLI, adult C57/BL6 adult mice (8 weeks) were used. They received an overdose of sodium pentobarbital (0.09 mg/g, i.p.) and were perfused transcardially with phosphate-buffered saline (0.1 M PBS) followed by 4% paraformaldehyde in 0.1 M PB. Their brains were then removed and postfixed in 4% paraformaldehyde for 24 h. Vibratome parasagittal sections (200 μm) of the cerebellum were obtained. Sections were prelabeled with 4, 6-diamidino-2-phenylindole (DAPI; Sigma, St Louis, MO), and a continuous current was used to inject with Lucifer yellow (8% in 0.1; Tris buffer, pH 7.4; LY) individual cells at different distances from the pial surface in the ML of the cerebellum. Following the intracellular injections, sections were processed for immunofluorescence staining. They were incubated for 72 h at 4 ºC in stock solution (2% bovine serum albumin, 1% Triton X-100, and 5% sucrose in PB) containing rabbit anti-LY (1:400,000; generated at the Cajal Institute, CSIC, Spain). The sections were then rinsed in PB and incubated in biotinylated donkey anti-rabbit IgG (1:100; Amersham, Buckinghamshire, United Kingdom). Then, sections were then rinsed again and incubated with Alexa fluor 488 streptavidin-conjugated (1:1000; Molecular Probes, Eugene, OR, United States of America). Finally, the sections were washed and mounted with ProLong Gold Antifade Reagent (Invitrogen Corporation, Carlsbad, CA, USA).

For cell reconstruction and quantitative analysis, sections were imaged with a confocal scanning laser attached to a fluorescence microscope Zeiss (LSM710). Consecutive stacks of images at high magnification (× 63 glycerol; voxel size, 0.057 × 0.057 × 0.14 μm^3^ for mouse cells) were acquired to capture dendritic, and in some cases also axonal, arbors on the basis of Lucifer yellow immunostaining.

Data points of neuron morphology of each ML interneuron were extracted in 3D using Neurolucida 360 (MicroBrightfield). Briefly, dendrites, axon and soma, in the skeleton definition were described through 3D points delimiting the different segments that form the cell arbor. These points have an associated diameter that provides the information of the varying thickness of the dendritic or axonal processes and varies along the length of the processes. The soma was defined through a set of connected points tracing the contour of the soma in 2D. Morphological variables were extracted using Neurolucida software.

### Computational modeling

**Figure 1:**
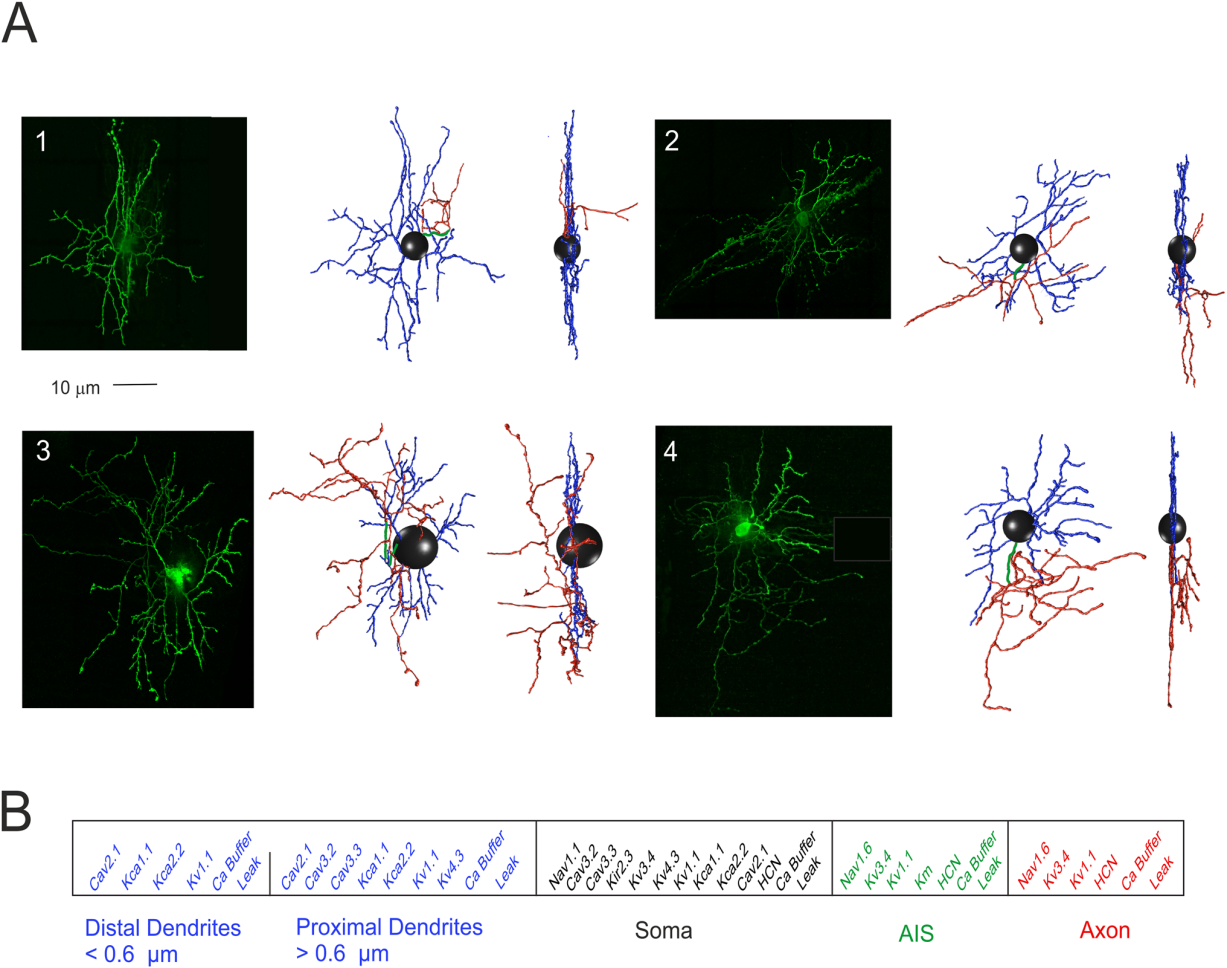
Stellate cell morphological reconstruction. **(A)** Confocal microscopy imaging of four mouse SCs filled with Lucifer yellow (scale bar 10 μm) are shown along with the corresponding digital reconstruction with Neurolucida (visualization with Vaa3D simulator; right). The 3D morphological reconstructions of SCs include dendrites (blue), soma (black), AIS (green) and axon (red). **(B)** The SC model is divided into five electrotonic compartments and endowed with specific ionic mechanisms according to immunohistochemical data. The proximal and distal dendrites are distinguished at the cut-off diameter of 0.6 µm. Ionic channels include Na^+^, K^+^ and Ca^2+^ channels and a Ca^2+^ buffering system.

In this work, we have constructed and simulated multi-compartment SC models, using Python-NEURON (Python 2.7.15; NEURON 7.7)^43^. The models were based on four mouse SC detailed morphologies. The ionic channels were distributed over the somatic, dendritic and axonal compartments, according to immunohistochemical, electrophysiological and pharmacological data, and modeled following the HH formulation or Markov-chains for multi-state transitions, using mathematical methods reported previously^30–33,44^ (see Fig. 1).

The models were optimized with BluePyOpt^45^. At the end of the optimization process the models are provided by the fine interaction of 14 ionic channels for a total of 36 maximum ionic conductances (G_i-max_) parameters assigned in the different morphology sections (Supplementary Figs. 1–2 and Table 1). G_i-max_ parameters have been found by an automatic optimization procedure to obtain the ensemble of electrophysiological behaviours. Each channel needed a precise value of G_i-max_ to interact, in an optimal way, with all the other values to achieve the exact balance between the sophisticated ionic channel dynamics.

**Figure 2:**
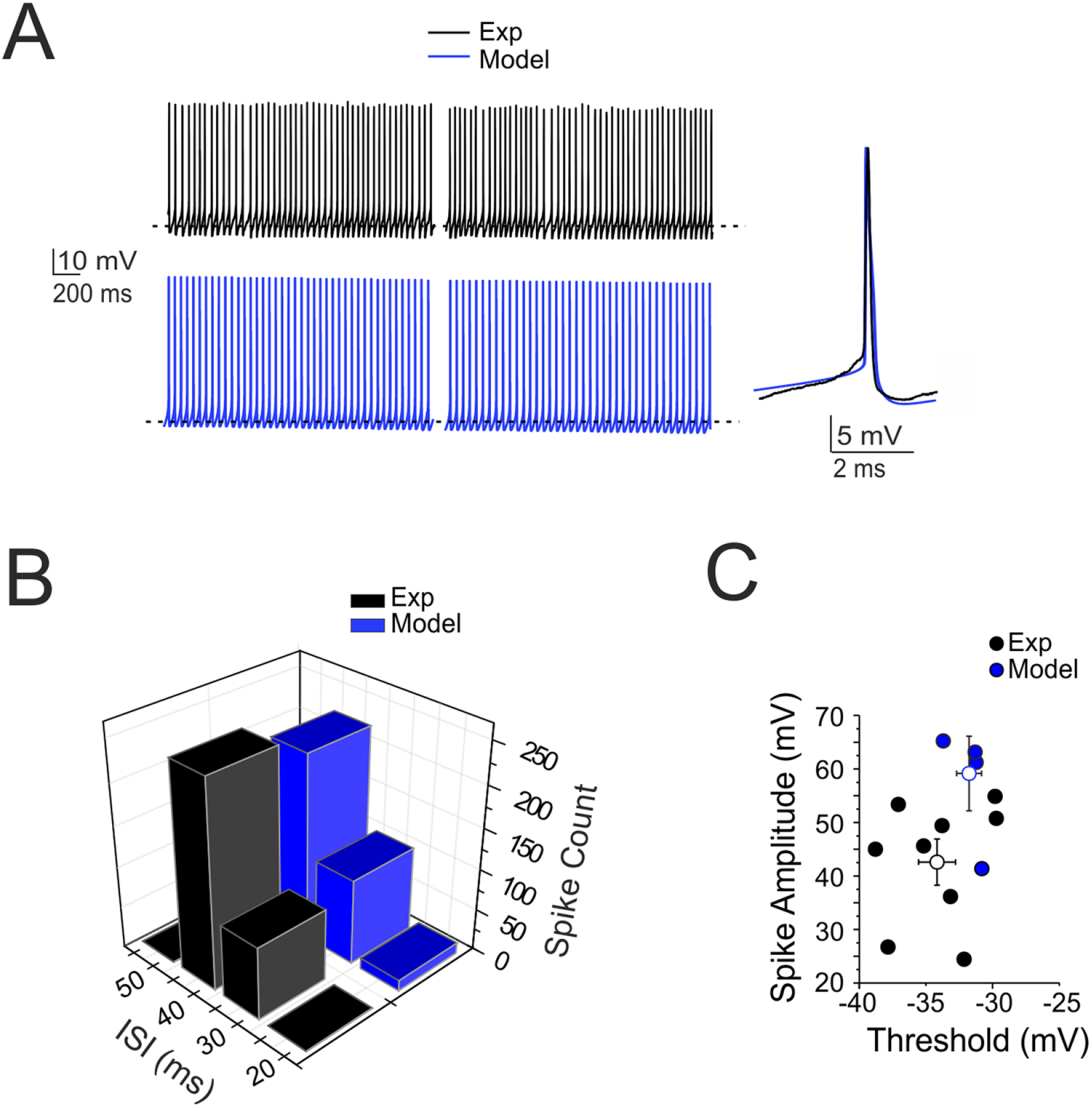
Pacemaking activity. **(A)** Pacemaker activity during SC WCR (n = 9; black) and in the model (n = 4; blue) taken at different times (traces taken starting at 2 s and 6 s). The inset shows a simulated and an experimental spike superimposed. **(B)** Distribution of the ISI of spontaneously firing SCs in WCR over 10 s. Note that the ISI in the model (blue bar; n = 4) falls within the experimental data distribution (black bar; n = 9, p = 0.8). **(C)** Relationships among ISI parameters. Note that the model data points (blue circles; n = 4) fall within the distribution of spike amplitude vs. spike threshold measured experimentally (black circles; n = 9). The model did not significantly differ from the experimental data (p = 0.64). Data are reported as mean ± SEM.

#### Morphology and synaptic location

Four mice stellate neurons were chosen from fluorescent images obtained with a confocal microscope and reconstructed with Neurolucida 360. The morphologies consisted of branched dendritic trees, a soma, and a branched axon with collaterals (the axon is short and unmyelinated). The table (Supplementary Table 2) shows the number of morphological compartments along with their diameter and length. The location of the inhibitory synapses on PCs was derived from available anatomo-physiological data. The PC dendrites are divided into three orders of branching receiving specific synaptic inputs (cf.^28^). Concerning excitatory synapses, branch II received only PF synapses and branch III received only ascending axon synapses^14,15,46^. Inhibitory synapses were distributed only on branches I and II for a total of 221 sections, since branches III were experimentally reported not to have inhibitory synapses^15,46^. Inhibitory synapses on branches I and II were made identical, although those on section I may have a different control^47^.

#### Passive properties and ionic channels

The passive properties of the SC model include R_axial_ which was set to 110 Ω cm for all the compartments, R_input_ = 0.75 ± 0.06 GΩ, C_m_ was set to 1.5 µF/cm^2^ for the dendrites while the rest of the compartments was set to 1µF/cm^2^. The reversal potential (E_rev_) of each ionic species was defined as follows: E_Na_ = 60 mV, E_k_ = − 84 mV, E_Ca_ = 137.5 mV, E_h_ = − 34 mV, E_leak_ = − 48 mV. The leakage G_i-max_ was set to 3e−5 S/cm^2^ for all the compartments. The SC ionic channel models and distributions were taken from previous papers and updated according to the latest literature when needed [see Supplemental Material for details;^33^]. The model included Na^+^ channels (Nav1.1 and Nav1.6), K^+^ channels (Kv3.4, Kv4.3, Kv1.1, Kir2.3, Kv7, KCa1.1 and KCa2.2), Ca^2+^ channels (Cav2.1, Cav3.2 and Cav3.3), hyperpolarization-activated cyclic nucleotide–gated channels (HCN1) and a parvalbumin-based Ca^2+^ buffer. It should be noted that most of ionic channel kinetics were measured from neurons other than SCs. This is a standard procedure since whole-cell voltage-clamp recordings are unpractical in most neurons with extended neurites, while high-quality ionic channel equations can be retrieved from reconstituted systems and are available from data-banks previous papers (for a discussion of the issue, see^33–35^).

#### Excitatory and inhibitory neurotransmission

SCs receive synaptic activity from excitatory pathways, such as the GrC axons and the diffusion from climbing fibers and local inhibitory pathways. The inhibitory activity of SC modules the spike transmission to PC. Several studies have proved the presence of specific types of AMPA and NMDA receptor-mediated currents in cerebellar SC^44,48,49^. AMPA and NMDA receptors, built with the Tsodyks formalism^50^, were used to simulate the excitatory synaptic activity from the PFs to SC dendrites.

The AMPA receptor received the following parameters^44^: release probability (*p*) = 0.15, recovery time constant (τ_R_) = 35.1 ms, facilitation time constant (τ_F_) = 10.8 ms, maximum ionic conductance (G_i-max_) = 2300 pS per synapse, ionic reversal potential (E_rev_) = 0 mV (Supplementary Fig. 3A and Table 3).

**Figure 3:**
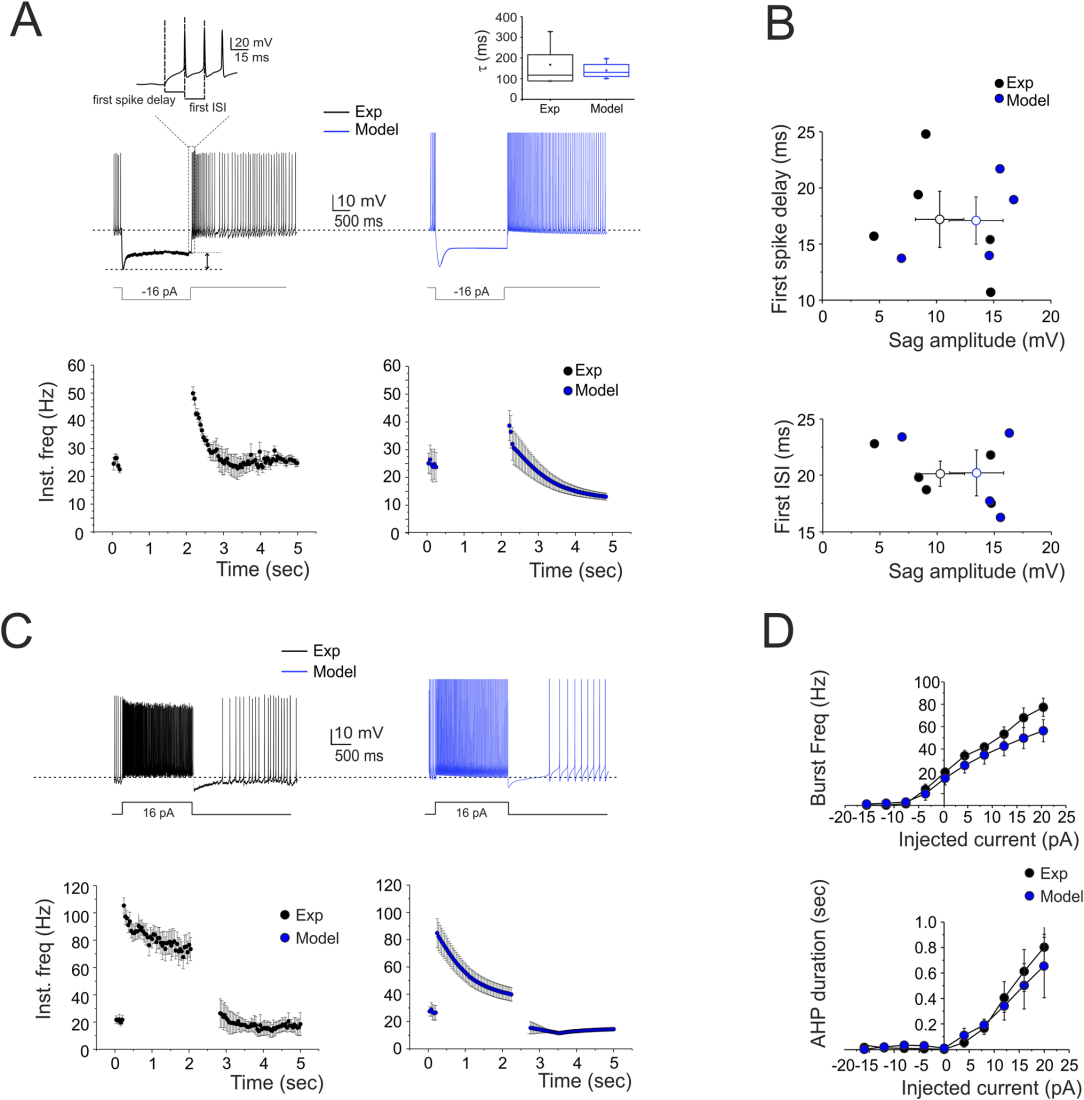
Response to hyperpolarization and depolarization. **(A)** A WCR from a SC shows sagging inward rectification in response to hyperpolarizing current injection and, at the end of the hyperpolarization, rebound excitation with an early and a protracted phase of intensified firing. Simulations of this specific experiment show that the model could faithfully reproduce sagging inward rectification and rebound excitation (blue trace). The enlarged trace on top shows how the first spike delay and the first ISI were determined. At the bottom, the time course of instantaneous spike frequency for a − 16 pA current pulse. The box-and-whisker plot compares the sag decay time constant obtained in experiments (n = 6) and simulations (n = 4) without revealing statistically significant difference (unpaired *t*-test, p = 0.4) **(B)** The plots report the time to first spike and the first ISI during rebound excitation as a function of sag amplitude (black circles; n = 5). Note that the model (blue circles; n = 4) did not significantly differ from the experimental data (p = 0.32). Data are reported as mean ± SEM. **(C)** A WCR from a SC shows the firing frequency and the pause following depolarizing current step (16 pA). The time course of instantaneous spike frequency for the 16-pA current pulse is shown below. Simulations show that the model could faithfully reproduce this behavior (blue traces). **(D)** In the plots, the response of the model (blue circles; n = 4) to current injections (from -16 to 20 pA) is compared to experimental data (black circles; n = 5). Simulations show that the model could appropriately fit the experimental measurements of spike frequency versus injected current (16 pA: p = 0.097) and of pause versus injected current (16 pA: p = 0.69). Data are reported as mean ± SEM.

The NMDA kinetic scheme was adapted to simulate the presence of the NR2B subunit^48^. NMDA NR2B receptor was modeled following the kinetics of the work of^51^ and the fitted parameters were: *p* = 0.15, τ_R_ = 8 ms, τ_F_ = 5 ms, G_i-max_ = 10,000 pS per synapse, E_rev_ = − 3.7 mV (Supplementary Fig. 3B and Table 3).

The GABA_A_ receptor model maintains the kinetic scheme described in the work of^52^ and modified by maintaining the α1 subunit but deleting the α6 subunit (absent in the MLI) as follows: *p* = 0.42, τ_R_ = 38.7 ms, τ_F_ = 0 ms, G_i-max_ = 1600 pS per synapse, E_rev_ = − 65 mV (Supplementary Fig. 3C and Table 3).

AMPA and NMDA receptors were placed on three distal dendrite compartments. GABA_A_ receptors were activated randomly on the dendrite compartments.

Model EPSCs and IPSCs were adapted to reproduce unitary synaptic current from SCs^23,44,52^ (Supplementary Fig. 3). The glutamatergic AMPA and NMDA receptors synaptic G_i-max_ parameter was balanced to reproduce a single PF EPSC, while *p* parameter was balanced to reproduce short-term facilitation and short-term depression (STF and STD) in SC EPSCs during a stimulus train.

#### Parameter optimization

In this work, we used an innovative technique that allows rapid and automatic parameter optimization, based on multi-objective evolutionary algorithms, called “Indicator-Based Evolutionary Algorithm” (IBEA)^53^ , in the BluePyOpt Framework^45^ , to estimate the maximum ionic conductance parameters (G_i-max_) (the free parameters of the model) of all ionic channels distributed along the morphology sections obtaining models able to reproduce the SC electroresponsiveness elicited by somatic current injections^40^.

The optimization workflow followed the same procedure used for parameters tuning in GrCs^34,35^. A set of 36 G_i-max_ values were tuned to match the firing patterns revealed by electrophysiological recordings. From the experimental traces, used as templates, we extracted the experimental features necessary to assess the fitness functions for the optimization procedure.

The following features, selected to better reproduce the biophysical properties of action potentials, were extracted using the ‘Electrophys Feature Extraction Library’ (eFEL)^54^ during the spontaneous firing and the current injection protocols: AP_Ampl_ (mV), AP_AHP_ (mV), AP_Thr_ (mV), (AP_HW_) (ms), AP_Freq_ (Hz) (Supplementary Tables 4 and 5).

Optimization tests were applied to have four different best models based on the four morphologies, starting the process with the same range of conductances. Tests were performed on Piz Daint Cluster (CSCS-Lugano), using two nodes for a total of 72 cores, with simulations of 2000 ms, fixed steps of 0.025 ms, temperature of 32 °C, as in the experimental recordings and all ionic current kinetics were normalized to this value using Q_10_ = 3 corrections^55^. The stimulation protocol included the spontaneous firing, two positive current injections (4, 16 pA) and a negative current injection (− 16 pA) lasting for 2 s. The optimizations involving 100 individuals for 50 generations, required 3 h of computation time.

It should be noted that, in principle, the spikes contain information relative to all the maximum ionic conductances of a neuron^28,33^. Indeed, model optimization using BluePyOpt^45^ was able to capture, starting from spike discharges elicited by somatic current injection, several unanticipated cellular properties including burst pause responses, either related to intrinsic excitability or to synaptic activation, that were further used for model validation.

#### Simulations and testing protocols

The best models yielded by the optimization process were simulated using protocols designed to reproduce the experimental ones. Each model was tested for: (i) the spontaneous firing frequency, (ii) the firing frequency obtained with positive current injections of 4 pA and 16 pA, (iii) the AHP after current injection and (iv) the sag generated by -16 pA current injection. The models were further tested with synaptic protocols. (v) PF activation was tested with synaptic trains composed by 10 pulses @ 4 Hz, 10 Hz, 20 Hz, 50 Hz, 100 Hz, 200 Hz, 500 Hz. (vi) GABAergic inhibitory activity, between two pair of SC, was tested using a background stimulation at 20 Hz with either 20, 27 or 32 GABA_A_ receptor-mediated synapses. The same PF trains were delivered in conjunction with the inhibitory background.

The SC model was also wired in a minimal microcircuit including PFs and a PC^28^. All the synapses involved were randomly distributed over the corresponding neuronal compartments. The PF formed 100 synapses on PC dendrites and 3 synapses on SC dendrites, the SCs formed 25, 50, 100, 200 or 300 synapses on PC dendrites. In a set of simulations, a second SC was connected through 32 synapses to the first one. SCs and the PC received PF stimuli made of 10 pulses @ 4 Hz, 10 Hz, 20 Hz, 50 Hz, 100 Hz, 200 Hz or 500 Hz.

The simulations were performed on an AMD Threadripper 1950X (32 GB ram), with fixed time step of 0.025 ms, temperature 32°. Simulation time was 2 s for spontaneous activity and current injections, 5 s to test PF-SC synaptic activity, 15 s to test the SC – > SC – > PC circuit.

#### Model validation

The model was validated at several levels by comparing its parameters to experimental measurements that were not used for construction. In particular, simulated spike amplitude and spike threshold (Fig. 2), spike delay, sag amplitude, and the first ISI (Fig. 3) and input–output gains during burst transmission (Fig. 6) were compared to experimental data without uncovering any statistically significant difference.

### Data analysis

Custom Python-scripts (Python 2.7.15; https://www.python.org) were written to automatically analyze the set of Gi-max parameters at the end of each optimization, to run simulations and to extract features. The voltage and current traces were analyzed using MATLAB 2018a/b (MathWorks, Natick, MA, USA; https://www.mathworks.com), pClamp 10 software (Molecular Devices, CA, USA; https://www.moleculardevices.com), OriginPro8 (https://www.originlab.com) software and MS Excel 2007. All figures were created with CorelDraw Graphics Suite 2018 software (https://www.coreldraw.com). The morphologies were analyzed with NEURON 7.7 (https://neuron.yale.edu) and visualized with Vaa3D (https://alleninstitute.org).

## Results

### Stellate cell physiology and modeling

Detailed models of SCs of mouse cerebellum were generated starting from morphological reconstructions and using electrophysiological recordings as a template for the optimization of membrane mechanisms^34,35^ (full explanation is given in Supplemental Material). Morphologies of four SCs were reconstructed from immunofluorescent images taken from fixed preparations ex vivo. WCR from 23 SCs were obtained in acute cerebellar slices. All neurons were selected in the outer two thirds of the molecular layer, where SCs are the only neuronal species present^6^.

The four reconstructed SCs corresponded to the canonical description reported in literature^3,6,56^. SCs showed a soma emitting 3–4 branched dendritic trees subdivided in proximal and distal sections, and axon initial segment (AIS) continuing in a branched axon (Fig. 1A). The soma surface measured 42.4 ± 9.3 µm^2^ (n = 4). Long, contorted and frequently branching dendrites extending from the soma were characterized centrifugally^57,58^, and quantified with two previously established measures^59^: total dendritic length (the summed length of all dendritic segments; 845.2 ± 121.2 µm; n = 4) and segment count (proximal dendrites: 11.0 ± 3.0, n = 4; distal dendrites: 63.5 ± 14.0, n = 4). Conversely, the axons branched immediately generating short and circumscribed collaterals (AIS length: 28.0 ± 6.7 µm, n = 4; axon length: 577.9 ± 225.0 µm, n = 4; Supplementary Table 2 and Movie 1). The dendritic tree was flattened on the sagittal plane of the *folium* and the axon, after an initial part parallel to the dendrite, advanced along the transverse plane^6,11,20^. The four morphologies were transformed into morpho-electrical equivalents (Fig. 1A) and used as the backbone for model reconstruction^33^. The SC models were divided into five electrotonic compartments and endowed with 14 different types of voltage-dependent and calcium-dependent ionic channels and with a calcium buffering system (Fig. 1B).

The electrophysiological properties of SCs were recorded from the cell somata of neurons located in the outer two thirds of the ML (Figs. 2, 3). The basic properties of SCs were homogeneous, according to anatomical indications^6^; see also above), and could be summarized as follows. (i) SC input resistance was 1.3 ± 0.1 GΩ; n = 23 and membrane capacitance was 6.7 ± 0.5 pF; n = 23. (ii) the SC spike was narrow (1.0 ± 0.2 ms, n = 9 (AP_HW_)) and overshooting (37.3 ± 5.2 mV (AP_Ampl_)) (an example is shown in Fig. 2A and detailed parameters are reported in Supplementary Tables 4 and 5). (iii) SCs fired spontaneous action potentials at 16–36 Hz (24.2 ± 2.1 Hz; n = 9; Fig. 2A). The average ISI distribution showed a single peak at 45.4 ± 3.5 ms (n = 9; Fig. 2B). There was no correlation between spike amplitude and threshold (Fig. 2C). (iv) When injected with hyperpolarizing current steps, SCs showed sagging inward rectification and, after hyperpolarization, showed rebound excitation (Fig. 3A). Rebound excitation consisted of accelerated spike frequency, which progressively decayed back to baseline. There was no correlation between the amplitude of the hyperpolarizing sag (10.3 ± 2.0 mV; n = 5), the first spike delay (17.2 ± 2.3 ms; n = 5) or the first ISI (20.1 ± 1.0 ms; n = 5) (Fig. 3B). (v) When injected with depolarizing current steps, SCs generated fast repetitive spike discharge. The output frequency increased almost linearly with current intensity (slope 2.79 Hz/pA; n = 5) (Fig. 3C,D). (vi) At the end of spike discharges elicited by step current injections, SCs showed a marked AHP delaying the re-establishment of pacemaker activity. The AHP duration increased with stimulation intensity (Fig. 3D).

The models (see “Methods” and Supplemental Material) faithfully reproduced all the properties reported above, as shown in Figs. 2 and 3. The statistical comparisons reported below demonstrate the absence of significant differences between the data obtained in WCR and the SC models. In detail, the model (i) matched the passive neuron properties, (ii) reproduced spike shape (Supplementary Tables 4 and 5), (iii) showed an average pacemaker frequency within the experimental data distribution (Fig. 2A,B; Supplementary Movie 2), (iv) responded to negative current injections, reproducing sagging inward rectification and rebound excitation (Fig. 3A), (v) increased firing rate with an almost linear I/O relationship in response to step current injections (slope 2.13 Hz/pA; n = 4) (Fig. 3D), (vi) showed a hyperpolarization followed by a pause in spike firing at the end of the current steps (Fig. 3D). The parameters of the four SC models corresponding to these electrophysiological properties fell within the experimental data distributions (p > 0.1, unpaired *t*-test), indicating that SCs electrical responses recorded in experiments and models could be treated as members of the same statistical distribution. The electroresponsive mechanisms of the models are shown in Supplementary Figs. 1 and 2 and quantified in Supplementary Fig. 4, highlighting the specific involvement of [Ca^2+^]_i_ changes, Cav3.2, KCa1.1 and other voltage-dependent ionic channels operating in SCs.

**Figure 4:**
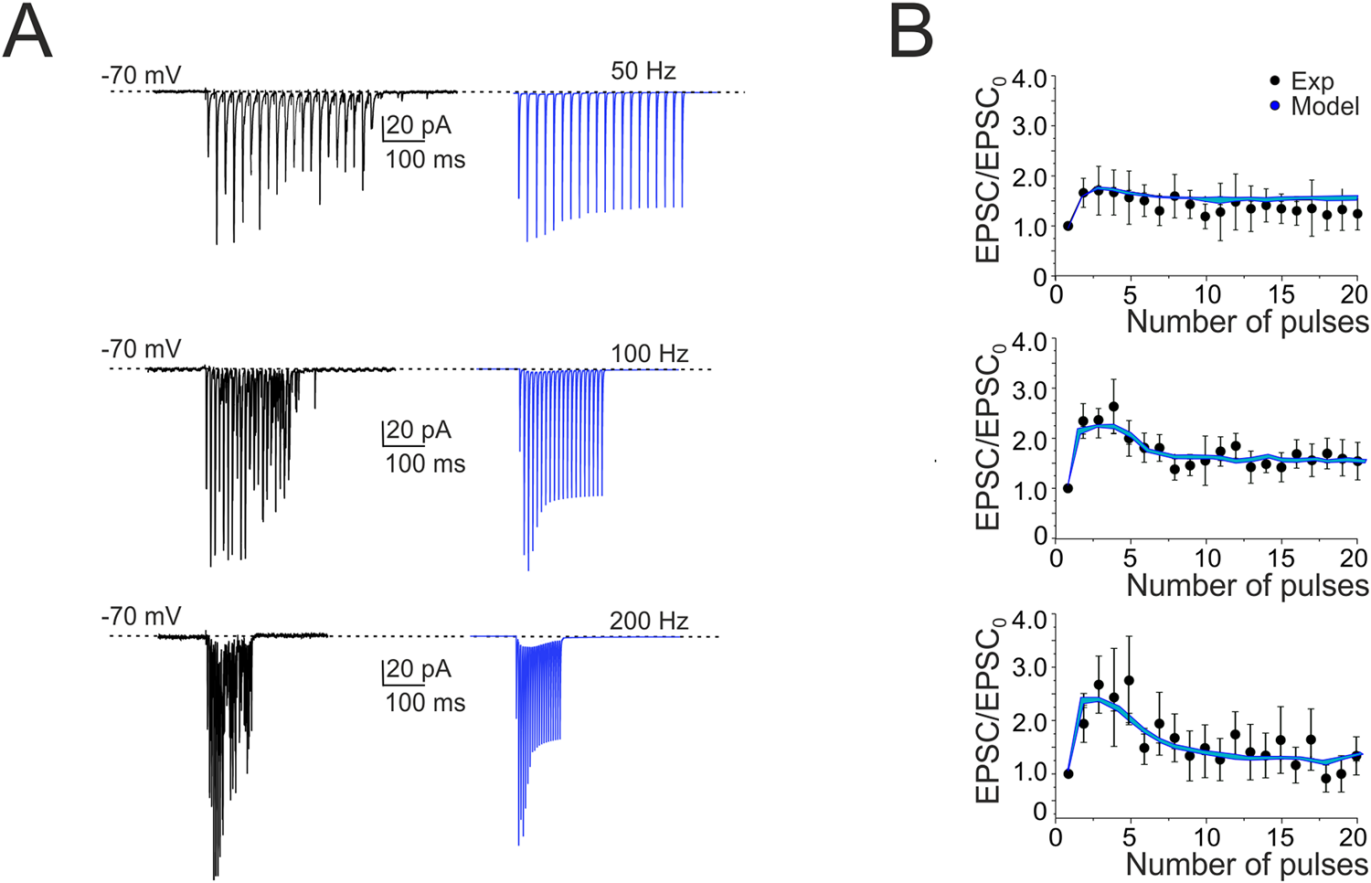
Short-term plasticity at PF-SC synapses. **(A)** EPSCs recorded from the SC during 50, 100 and 200 Hz parallel fiber stimulations (n = 6). Cells were voltage-clamped at − 70 mV and in the presence of gabazine. Each trace (black) is an average of 10 sweeps. Note that EPSC trains showed first facilitation and then depression. Simulations show that the model could faithfully reproduce the same behavior (blue traces; n = 4). **(B)** Comparison of peak amplitudes of experimental and simulated EPSCs during high-frequency parallel fiber stimulation. Amplitudes are normalized to the first EPSC in the train (correlation coefficient R^2^ @ 50 Hz = 0.80; @ 100 Hz = 0.90; @ 200 Hz = 0.84). Data are reported as mean ± SEM.

### Frequency-dependent short-term dynamics at parallel fiber-stellate cell synapses

In order to understand how information transmitted by GrC inputs is encoded by SCs, we investigated response dynamics at PF-SC synapses activated by trains of 20 PF stimuli at different frequencies (50, 100 and 200 Hz), to preserve PFs, these experiments were performed in coronal slices (see “Methods”). During the stimulus trains, EPSCs showed an initial strong facilitation followed by depression, which however did not make the response smaller than control (Fig. 4A,B)^60–62^. The sequence of STF and STD was the more evident the higher the stimulation frequency, suggesting that frequency-dependent short-term synaptic dynamics could regulate transmission efficacy along the PF-SC pathway^17^.

**Figure 5:**
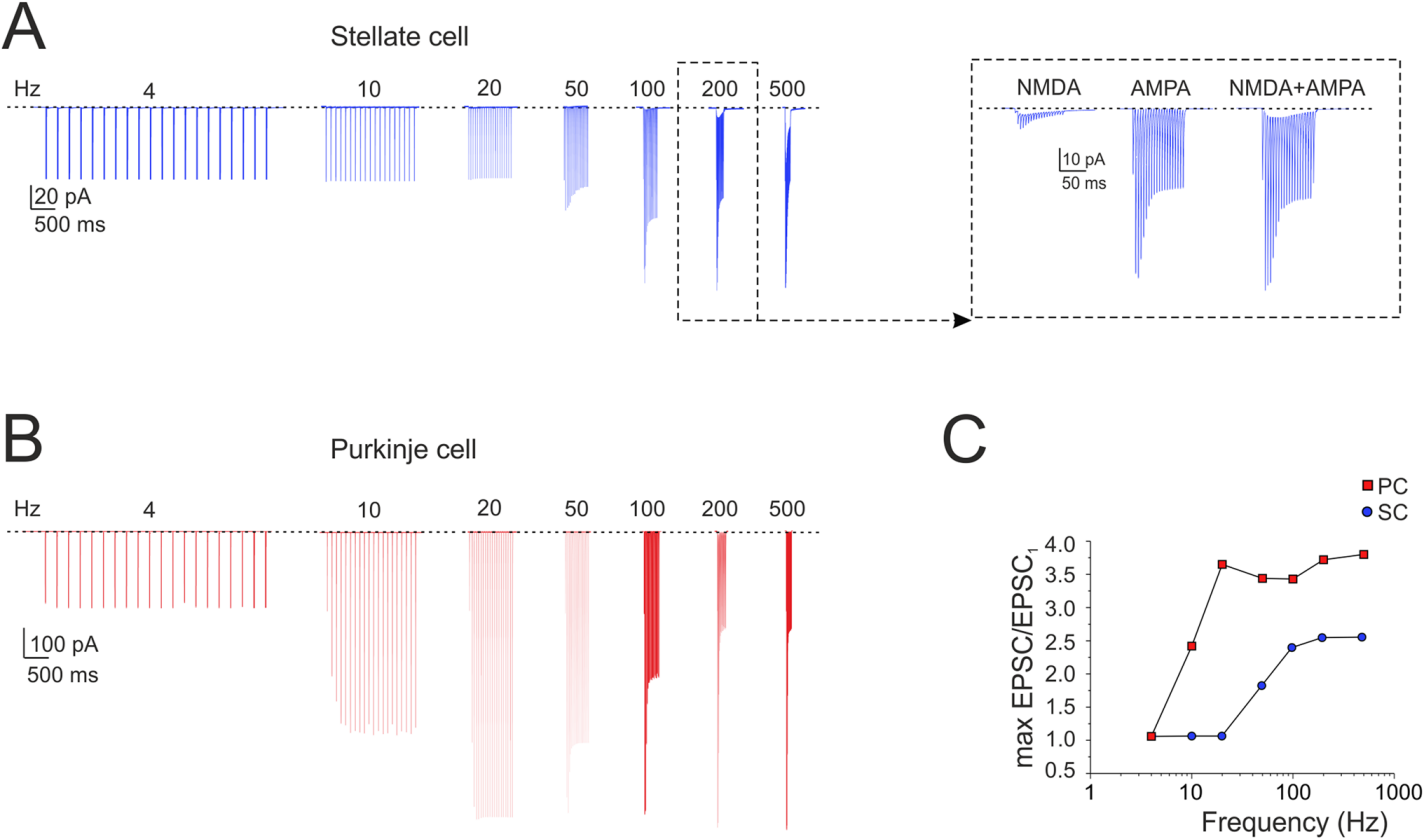
Burst currents at PF-SC and PF-PC cell synapses. **(A)** The traces show simulated SC EPSCs during trains of 20 stimuli delivered to PFs at different frequencies (4, 10, 20, 50, 100, 200 and 500 Hz; n = 4). Inset, NMDA, AMPA and NMDA + AMPA currents at 200 Hz. **(B)** The traces show simulated PC EPSCs during trains of 20 stimuli delivered to PFs at different frequencies (4, 10, 20, 50, 100, 200 and 500 Hz). **(C)** Gain curve for SC (blue circles; n = 4 for each frequency) and PC (red squares) responses with respect to input burst frequency. Gain is the ratio between the maximum response obtained at a certain frequency and the first EPSC. The gain curves show a sigmoidal shape with 50% amplitude around 50 Hz for the SC and around 10 Hz for the PC. Data in C corresponds to the cells in A and B.

The PF-SC model of synaptic transmission^44,50^ was tuned to follow the experimental results yielding release probability, *p* = 0.15^44,48,49^. The low *p* level generated STF, while vesicle depletion during the train caused the subsequent STD (Fig. 5A). The model precisely followed the experimental results (cf. Figure 4B). The NMDA receptors enhanced PF-SC synaptic transmission (Fig. 5A inset; see also Fig. 7B).

For comparison, we have run a similar simulation on a PC model^28,33^, resulting in a similar sequence of STF and STD but with different frequency-dependence (Fig. 5B). The different frequency dependence was related to the different release probability and the different time constants determining short-term plasticity dynamics (see Supplementary Table 3). This observation suggested that the two cells, if wired together, would generate specific filtering effects (see below). The gain curve with respect to input burst frequency showed a sigmoidal shape with 50% amplitude around 50 Hz for the SC and around 10 Hz for the PC (Fig. 5C).

### Frequency-dependence of stellate cell input–output gain functions

**Figure 6:**
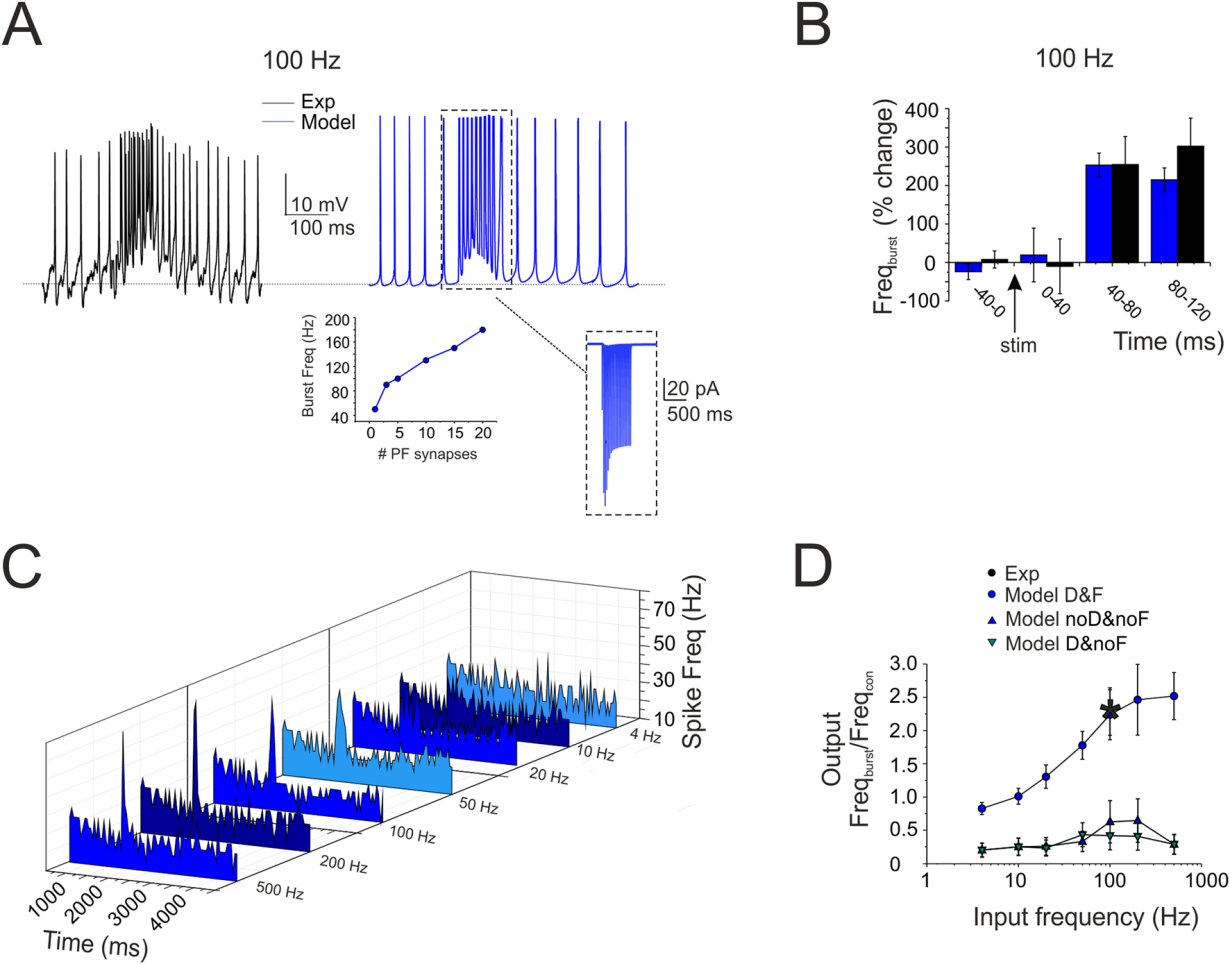
Frequency-dependence of SC input–output gain function. **(A)** The traces show a SC burst in response to 10 pulses @ 100 Hz-delivered to PFs (black trace) and the corresponding simulation (blue trace). Inset (left), calibration of the number of PF-SC synapses required to obtain a given burst frequency (3 in the example). Inset (right), the synaptic current (AMPA + NMDA) corresponding to the simulated burst. **(B)** The histogram shows the time course of the SC burst shown in A in response to 10 pulses @ 100 Hz-delivered to PFs (black trace) and the corresponding simulation (blue trace). Note the about 50 ms delay to burst response. **(C)** The array of PSTHs shows the SC responses to PF bursts at different frequencies (10 pulses @ 4 Hz, 10 Hz, 20 Hz, 50 Hz, 100 Hz, 200 Hz, 500 Hz). Note that pronounced spike bursts were generated at high frequencies (e.g. at 100 Hz). (D) Input/output SC gain for experimental (black, n = 5) and simulated bursts (blue, n = 4). SCs did not increase their spike output frequency until about 10 Hz, then their responses increased and tended to saturate beyond 100 Hz. Note the superposition of the single experimental data point and simulated data (asterisk). The simulation was repeated after the switch-off of STF (τ_facil_ = 10 times the original) and STD (τ_rec_ = 0), revealing their critical role for the frequency-dependence of the input–output function. Data in B and D are reported as mean ± SEM (n = 4).

The impact of short-term dynamics at PF-SC synapses was investigated by measuring the responses to PF bursts at different frequencies (10 pulses @ 4 Hz, 10 Hz, 20 Hz, 50 Hz, 100 Hz, 200 Hz, 500 Hz). Pronounced spike bursts were generated at high frequencies (e.g. at 100 Hz, the frequency increase was 154.5 ± 40.5%, n = 4; p = 0.046; Fig. 6A). A careful analysis of PSTHs showed a delay in the first spikes of SCs in response to high-frequency PF stimulation (about 50 ms at 100 Hz) probably determined by STF in synaptic transmission (Fig. 6B). The analysis of responses to different PF input frequencies revealed that SC responses did not increase their spike output frequency above the basal frequency below about 10 Hz, then their responses increased and tended to saturate beyond 100 Hz (Fig. 6C). The input/output gain curve showed a sigmoidal shape (Fig. 6D). This effect was related to STF, since it disappeared when STF was switched off (Fig. 6D).

The models were calibrated by activating an increasing number of synapses at 100 Hz, reveling that an output frequency like the experimental one was obtained using just 3 PF-SC synapses (Fig. 6A, inset). The experimental data were then faithfully reproduced by the models (Fig. 6 A-D; see also Supplementary Fig. 3) by simply using the electroresponsiveness and synaptic transmission properties calibrated beforehand, thus providing a mechanistic explanation for the time-dependent and frequency-dependent properties of SCs synaptic responsiveness.

**Figure 7:**
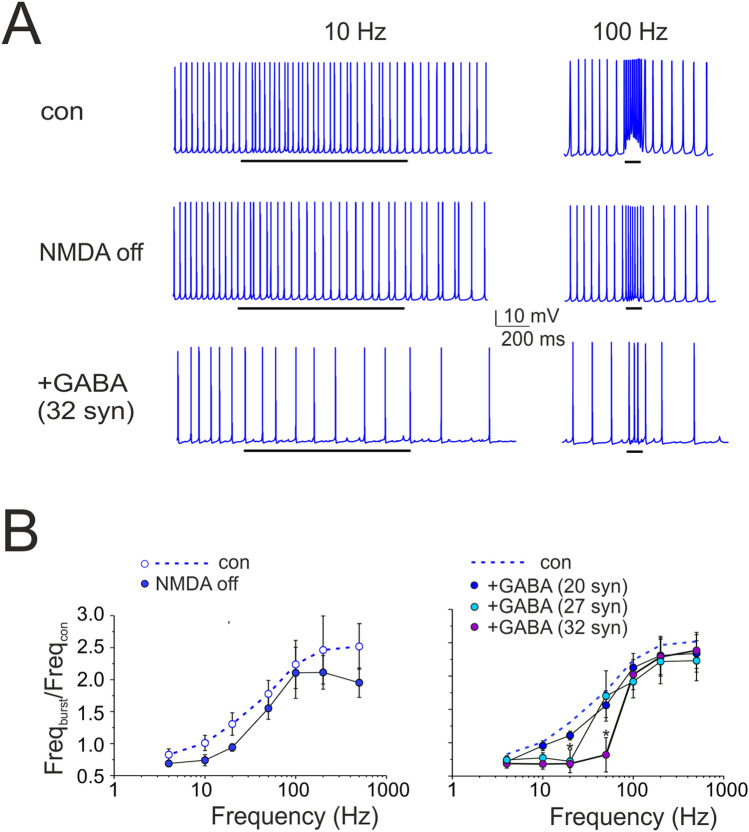
Regulation of SC gain function by NMDA and GABA_A_ receptors. **(A)** The traces show simulated SC response to 10 pulses @ 100 Hz-delivered to PFs. NMDA receptor switch-off and activation of inhibitory (32) synapses activated simultaneously with the PF burst reduced SC firing rate. Black bar indicates the stimulus duration. **(B)** Input/output SC gain regulation. Note that the NMDA current block reduced the spike output frequency mostly during high-frequency bursts (200–500 Hz), while a sufficient number of inhibitory synapses (activated simultaneously with PF bursts) shifted SC excitation toward higher input frequencies. Data are reported as mean ± SEM (n = 4).

The model was further used to evaluate the effect of factors that could modulate the gain curve. NMDA current stimulation^49,63^;^64^ blockage reduced (though not significantly) the SC firing rate during high-frequency bursts (e.g. at 200 Hz, frequency change was − 21.4 ± 9.2%, n = 4; p = 0.13; Fig. 7A, B). Of special interest was the simulated effect of inhibitory GABA_A_ receptor-mediated synaptic transmission from neighboring SCs^42,52,65,66^. To estimate the strength of this connectivity, a series of simulations were performed with different numbers of inhibitory synapses activated simultaneously with PF bursts. While increasing the inhibitory strength, SC excitation was moved toward higher frequencies and the activation curve became steeper (50 Hz _(mod)_: − 70.9 ± 9.4%, n = 4; p = 0.009; Fig. 7B). Dendritic voltage-dependent currents activated during repetitive synaptic stimulation of the SC model are considered in Supplemental Material (Supplementary Fig. 5; compare the currents elicited by step current injection in Supplementary Fig. 4). In conclusion, during PF stimulation, NMDA receptor modulation was more evident at higher frequencies whereas GABAergic modulation was more evident at lower frequencies suggesting that two synaptic systems had opposite effects on the SC gain function.

### Prediction of stellate cell filtering of Purkinje cell responses

**Figure 8:**
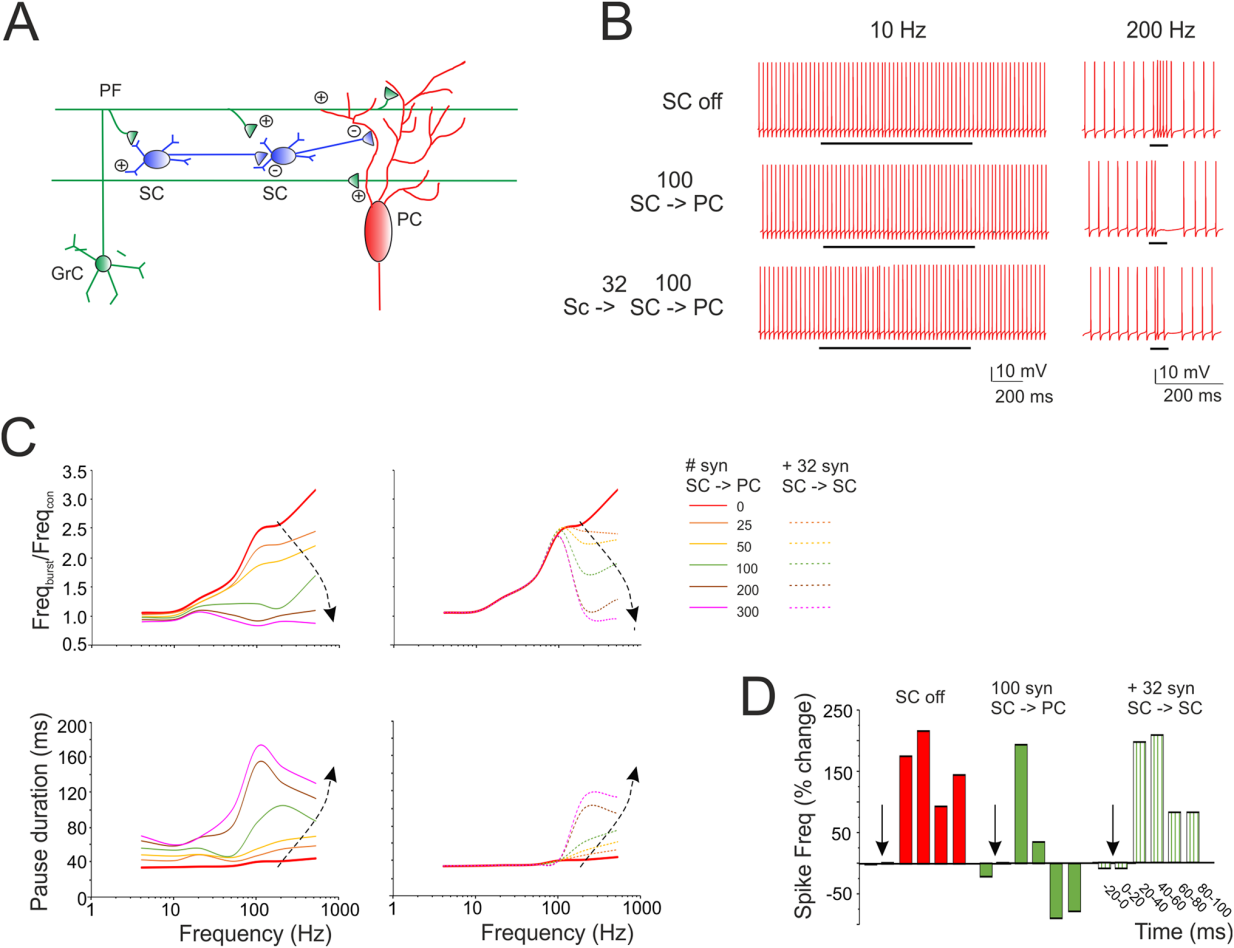
Prediction of SC filtering of PC responses along the PFs. **(A)** Schematics of the afferent connections to a PC activated by PF stimulation. Granule cell (GrC); parallel fiber (PF); stellate cell (SC); Purkinje cell (PC). The figure highlights the interactions of elements in the cerebellar molecular layer and the location of afferent PC synapses. **(B)** The traces show simulated PC response to 10 pulses at different frequencies (10, 200 and 500 Hz) delivered to 100 PFs in which (i) SCs were not activated (SC off), (ii) 100 SC synapses were activated (SC – > PC) and (iii) 100 SC synapses received inhibition from 32 SC synapses (SC – > SC – > PC). Black bar indicates the stimulus duration. **(C)** Input/output PC burst frequency gain (top) and pause length (bottom). Different curves are obtained using PF trains at different frequency (10 pulses @ 4 Hz, 10 Hz, 20 Hz, 50 Hz, 100 Hz, 200 Hz, 500 Hz) and an increasing number of inhibitory synapses. Dotted traces also include the case of SC-SC inhibition. Note that PC burst and pause showed an almost opposite modulation by SCs. **(D)** The histogram shows the regulation of PC firing frequency when SCs are off, on and reciprocally inhibited. Note that reciprocal SC inhibition can abolish the effect of SCs on PCs.

SCs provide feed-forward inhibition to PCs^14,15,28^. Here, the different frequency-dependence of SC and PC responses to input bursts suggested that SCs could act as filters, when the two cells are co-activated (Fig. 8A).

To explore the issue, we performed simulations in which SCs were synaptically connected to PCs (Supplementary Movie 3, 4). When SC synapses were activated, the PC was inhibited and its output spike frequency decreased. The effect was stronger at high input frequencies, where SCs are more activated. Consequently, a sufficient number of SC synapses (> = 50 Hz) abolished the response of PCs at high frequency (> 50 Hz) without affecting that at low frequency (< 50 Hz), de facto flattening the gain curve. In this configuration, the SC-PC system acted as an effective low-pass filter (Fig. 8B,C).

SCs are wired together through inhibitory synapses. We therefore performed simulations in which, in addition to stellate-PC feed-forward inhibition, SCs were reciprocally inhibited. In this case, the effect on the PC gain curve changed. Reciprocal inhibition reduced SC activity above 50 Hz, while leaving the PC excited above 10 Hz (cf. Fig. 5C). In this configuration, the SC – > SC – > PC system acted as low-pass filter was partially deactivated (Fig. 8B,C).

PCs normally generate burst-pause responses following PF bursts^28,67^. The simulations showed that, along with the burst, the SCs also regulated the pause although in an opposite manner (Fig. 8B,C). The pause was almost insensitive to input frequency but increased remarkably along with SC inhibition. It should be noted that the pause persisted even when PF stimulations lasted hundreds of milliseconds, as it happens in certain behavioral tasks like eye-blink classical conditioning^68^ (Supplementary Fig. 5A). Finally, SCs activity delayed PC responses by about 20 ms (Fig. 8D). In aggregate, these simulations show that SCs exert a remarkable effect on the delay, band-pass and burst-pause behavior of PCs.

## Discussion

This work provides the first detailed models of a small set of cerebellar SCs. The models are based on precise morphological and electrophysiological measurements and faithfully reproduce the non-linear excitable properties and short-term neurotransmission dynamics at the PF–SC synapse. The models give insight into the critical role that these neurons could play in molecular layer processing. By exploiting membrane excitability and short-term synaptic plasticity, SCs recode PF bursts and regulate the gain of PCs in a frequency-dependent manner. SC inhibition of PCs occurs above 10 Hz and, depending on the engagement of SC-SC inhibitory chains, can generate low-pass or band pass filters. These observations expand the concept of the cerebellum as an adaptive filter^41^.

### Stellate cell electroresponsiveness and synaptic regulation

SCs showed autorhythmic firing around 25 Hz^8,24^. In slice preparations, PFs and climbing fibers are inactive^69^, and the only connection conveying spontaneous activity comes from other SCs and basket cells. It is thus probable that SC pacing reflected intrinsic electroresponsive properties since, in our recordings, GABAergic inputs were blocked pharmacologically. When stimulated with positive or negative currents, SCs showed burst-pause or pause-burst responses resembling those of other cerebellar neurons, comprising PCs^28,38^, Golgi cells^31,32,70^ and deep nuclear cells^71,72^. Therefore, SCs are well suited to take part into the complex bursting and pausing dynamics of the cerebellar circuit^67^.

While several ionic channels have been reported in these neurons, a couple of them revealed their fundamental role in burst-pause dynamics of SCs (see Supplementary Figs, 1, 2 and 4). HVA-type channels (Cav2.1) were critical to regulate [Ca^2+^]_i_ and KCa1.1, thereby determining adaptation and pauses after bursts. LVA-type channels (mostly Cav3.2) allowed to generate rebound bursting after a prolonged hyperpolarization. H-type channels (HCN1) activated during hyperpolarization and could regulate pause duration, while A-type channels (Kv4.3) were most active during rebound bursts and could regulate spike delay. The same ionic channels were activated during patterns of repetitive synaptic transmission composed of excitatory and inhibitory bursts (see Supplementary Fig. 5).

Synaptic currents reflected the specific receptor-channel properties reported in voltage-clamp recordings^44,49,51,52^ and, when coupled to a presynaptic release mechanism, faithfully accounted for repetitive neurotransmission in bursts at different frequencies. While AMPA receptor-mediated currents generated rapid EPSCs and fast membrane depolarization, NMDA currents caused a slower build-up of the response during the trains. GABA_A_ receptors generated SC inhibitory currents. Interestingly, the kinetics of synaptic response trains showed strong facilitation (STD followed the first impulses at > 50 Hz) that, in the model, implied a low neurotransmitter release probability (*p* = 0.15). Consequently, the SCs generated spikes with a delay (about 50 ms during a 100 Hz train) and prevented responses to single PF spikes. This mechanism therefore effectively controls the timing of SC responses to bursts at the same time preventing their activation by single sparse PF spikes, which are therefore filtered out as noise.

As long as the models predict that SCs operate as delay-high-pass filters, they also anticipate the underlying mechanisms. The SC gain curves were enhanced by NMDA and reduced by GABA_A_ receptor-mediated transmission, this latter also shifting the cut-off frequency from ~ 50 Hz to ~ 100 Hz. The frequency-dependent effect reflected the short-term synaptic dynamics typical of these neurons and were enhanced by activation of NMDA currents and voltage-dependent ionic currents (mostly the LVA calcium current). GABA_A_ currents reduced the cell input resistance and prevented regenerative depolarizing effects (see also Supplemental Material).

### Stellate cell regulation of Purkinje cell gain

The electrophysiological and imaging analysis of signal propagation and neuronal excitation^17–19,63^ has revealed that granular layer signals are retransmitted to PCs vertically at high frequency along GrC ascending axons and then transversally at low frequency along PFs^73^. The present simulations indeed provide a plausible mechanism for this dual nature of transmission properties through the granular and molecular layer. This view substantially differs from the classical “beam theory”^4,74^, which maintained a central role for beams of PCs formed along the PFs. We cannot predict whether these PC beams will form or not, but we can anticipate that the PCs lying along the PFs receive a low-pass filtered signal from the PF– SC subcircuit that seems more suitable to synchronize them on the theta band than to perform actual information transfer from granular to molecular layer.

As well as SCs, also PCs showed STF and their gain increased with input frequency (cut-off ~ 20 Hz). When the SC and PC models were put in series along the PF beam so as to emulate a feed-forward inhibitory circuit^14,15,28^, the gain of PCs was limited to the values typical of low-frequency transmission (4–10 Hz) generating a low-pass filter. When the SC–SC feed-forward inhibition^10,11^ was activated, the gain curve of the PC showed a decrease beyond 100 Hz, generating a band-pass filter.

Validation of these simulations is provided by experiments that were performed using voltage-sensitive dyes in coronal slices. While PCs on-beam showed increased gain at high frequency when GABA_A_ receptors were blocked, their responses were low-pass filtered to the 4–10 Hz range when synaptic inhibition was active^17^. In addition to this, spots of PCs receiving activation along vertical beams, proved capable of high-frequency gain probably reflecting the exceeding number of GrC ascending axon synapses on PCs compared to the low number of PF synapses on local SCs^15,18^. Thus, while evidence for low-pass filtering is available, that for band-pass filtering awaits experimental validation.

An interesting observation emerging from these simulations is the relatively high number of SC synapses required to inhibit PCs, which exceeds that anticipated by the SC/PC ratio obtained from morphological measurements^75^. Indeed, with 100 synapses we appropriately reproduced low-pass filtering^17^ and controlled PC firing as in the original observation of Eccles^4^. Importantly, the physiological effect of inhibition was analyzed in single cell models^28,76^ that already suggested that hundreds of SC synapses were required to effectively regulate PC discharge^77^. In aggregate, our simulations suggest that the number of inhibitory SC-PC synapses is higher than previously thought, opening an interesting question for future anatomo-physiological studies.

### The molecular layer as an adaptive filter

A main prediction of the present simulations is that the intensity and bandwidth of molecular layer filtering is modulated by the number of active synapses between PFs, SCs and PCs. The number of active synapses, in turn, would depend on the organization of spike discharge in the PFs, and therefore, ultimately, on the reaction of the granular layer to the mossy fibers input^78–80^. Moreover, in addition to PF-PC synapses^81–85^, also the synapses between PFs and SCs^86–88^ and between SCs and PCs^89–91^ have been reported to develop long-term synaptic plasticity [for a recent review see^92–94^]. It is therefore possible that the PF-SC-PC system operates as a bank of filters, in which gain and bandwidth of PC responses are fine-tuned by SCs. Interestingly, mechanisms of signal filtering tuned by local synaptic plasticity have recently been recognized also in the cerebellum granular layer^35,95^ extending the mechanisms that could actually transform the cerebellum into an adaptive filter^41^.

## Conclusions

In conclusion, SCs emerge as critical elements controlling cerebellar processing in the time and frequency domain. In combination with VSD recordings^17^, the present simulations support the view that computation along PFs is different from that occurring in the spots of PCs laying vertically on top of active granular layer clusters^63,96,97^. Tuning of transmission bandwidth and delay through specific membrane and synaptic mechanisms may contribute to explain the role of SCs in motor learning and control^16^. For example, transmission of low-frequency bursts along the PFs may help tuning the molecular layer with cortical low-frequency oscillatory inputs on the theta-band^98,99^, with reflections on circuit synchronization and on the induction of long-term synaptic plasticity. These potential consequences remain to be investigated using advanced recording techniques and large-scale circuit simulations^39,100^.

## Supplementary Information

Supplementary Video 1.

Supplementary Video 2.

Supplementary Video 3.

Supplementary Video 4.

Supplementary Information.

## Data Availability

The experimental traces can be found on the HBP knowledge Graph at this link: https://kg.ebrains.eu/search/instances/Dataset/3ca4af33-64bd-437a-9c53-2dac19e10168.The models will be made available on the Brain Simulation Platform (BSP) of the Human Brain Project (HBP) as a “live paper” containing a selection of routines and optimization scripts. A wider selection will be made available on the HBP knowledge Graph. The models will also be uploaded on ModelDB.

## References

[1] Golgi, C. in *Archivio Italiano per le Malattie Nervose e più particolarmente per le Alienazioni Mentali* Vol. 11 90–107 (1874).

[2] Cajal, S. The Croonian Lecture. La fine structure des centres nerveux. *Proc. R. Soc. Lond.* **55**, 444–468 (1894).

[3] Cajal S (1888). Sobre las fibras nerviosas de la capa molecular del cerebelo. Rev. Trim. Histol. Norm. Patol..

[4] Eccles, J., Ito, M. & Szentagothai, J. *The Cerebellum as a Neuronal Machine | SpringerLink*, 10.1007/978-3-662-13147-3 (1967).

[5] Eccles JC (1967). Circuits in the cerebellar control of movement. Proc. Natl. Acad. Sci. U S A.

[6] Chan-Palay V, Palay SL (1972). The stellate cells of the rat's cerebellar cortex. Z. Anat. Entwicklungsgesch..

[7] Palay, S. & Chan-Palay, V. *Cerebellar Cortex: Cytology and Organization.* (1974).

[8] Hausser M, Clark BA (1997). Tonic synaptic inhibition modulates neuronal output pattern and spatiotemporal synaptic integration. Neuron.

[9] Jaeger D, Bower JM (1999). Synaptic control of spiking in cerebellar Purkinje cells: Dynamic current clamp based on model conductances. J. Neurosci..

[10] Mittmann W, Koch U, Hausser M (2005). Feed-forward inhibition shapes the spike output of cerebellar Purkinje cells. J. Physiol..

[11] Rieubland S, Roth A, Hausser M (2014). Structured connectivity in cerebellar inhibitory networks. Neuron.

[12] Wulff P (2009). Synaptic inhibition of Purkinje cells mediates consolidation of vestibulo-cerebellar motor learning. Nat. Neurosci..

[13] Jaeger D, De Schutter E, Bower JM (1997). The role of synaptic and voltage-gated currents in the control of Purkinje cell spiking: A modeling study. J Neurosci.

[14] Santamaria F, Tripp PG, Bower JM (2007). Feedforward inhibition controls the spread of granule cell-induced Purkinje cell activity in the cerebellar cortex. J. Neurophysiol..

[15] Bower, J. M. Model-founded explorations of the roles of molecular layer inhibition in regulating Purkinje cell responses in cerebellar cortex: more trouble for the beam hypothesis. *Front. Cell Neurosci.* **4**, 10.3389/fncel.2010.00027 (2010). 10.3389/fncel.2010.00027 PMC2944648 20877427

[16] Jorntell H, Bengtsson F, Schonewille M, De Zeeuw CI (2010). Cerebellar molecular layer interneurons—Computational properties and roles in learning. Trends Neurosci..

[17] Mapelli J, Gandolfi D, D'Angelo E (2010). High-pass filtering and dynamic gain regulation enhance vertical bursts transmission along the mossy fiber pathway of cerebellum. Front. Cell Neurosci..

[18] Cohen, D. & Yarom, Y. Cerebellar on-beam and lateral inhibition: Two functionally distinct circuits. *J. Neurophysiol.* **83**, 10.1152/jn.2000.83.4.1932 (2000). 10.1152/jn.2000.83.4.1932 10758104

[19] Cohen D, Yarom Y (1998). Patches of synchronized activity in the cerebellar cortex evoked by mossy-fiber stimulation: questioning the role of parallel fibers. Proc. Natl. Acad. Sci. U S A.

[20] Prestori, F., Mapelli, L. & D’Angelo, E. Diverse neuron properties and complex network dynamics in the cerebellar cortical inhibitory circuit. *Front. Mol. Neurosci.* **12**, 10.3389/fnmol.2019.00267 (2019). 10.3389/fnmol.2019.00267 PMC6854908 31787879

[21] ten Brinke, M. *et al.* Evolving models of Pavlovian conditioning: Cerebellar cortical dynamics in awake behaving mice. *Cell Rep.* **13**, 10.1016/j.celrep.2015.10.057 (2015). 10.1016/j.celrep.2015.10.057 PMC4674627 26655909

[22] ten Brinke, M. *et al.**Cell Rep.* **13**, 1977–1988 (2015). 10.1016/j.celrep.2015.10.057 PMC4674627 26655909

[23] Llano, I. & Gerschenfeld, H. Inhibitory synaptic currents in stellate cells of rat cerebellar slices. *J. Physiol.* **468**, 10.1113/jphysiol.1993.sp019766 (1993). 10.1113/jphysiol.1993.sp019766 PMC1143821 7504726

[24] Carter AG, Regehr WG (2002). Quantal events shape cerebellar interneuron firing. Nat. Neurosci..

[25] Molineux ML, Fernandez FR, Mehaffey WH, Turner RW (2005). A-type and T-type currents interact to produce a novel spike latency-voltage relationship in cerebellar stellate cells. J. Neurosci..

[26] Anderson D (2013). The Cav3-Kv4 complex acts as a calcium sensor to maintain inhibitory charge transfer during extracellular calcium fluctuations. J. Neurosci..

[27] Astori, S. & Köhr, G. Sustained granule cell activity disinhibits juvenile mouse cerebellar stellate cells through presynaptic mechanisms. *J. Physiol.* **586**, 10.1113/jphysiol.2007.146522 (2008). 10.1113/jphysiol.2007.146522 PMC2375591 18033809

[28] Masoli S, D'Angelo E (2017). Synaptic activation of a detailed Purkinje cell model predicts voltage-dependent control of burst-pause responses in active dendrites. Front. Cell Neurosci..

[29] Diwakar S, Magistretti J, Goldfarb M, Naldi G, D'Angelo E (2009). Axonal Na+ channels ensure fast spike activation and back-propagation in cerebellar granule cells. J. Neurophysiol..

[30] D'Angelo E (2001). Theta-frequency bursting and resonance in cerebellar granule cells: Experimental evidence and modeling of a slow k+-dependent mechanism. J. Neurosci..

[31] Solinas S (2007). Computational reconstruction of pacemaking and intrinsic electroresponsiveness in cerebellar Golgi cells. Front. Cell Neurosci..

[32] Solinas S (2007). Fast-reset of pacemaking and theta-frequency resonance patterns in cerebellar golgi cells: Simulations of their impact in vivo. Front. Cell Neurosci..

[33] Masoli S, Solinas S, D'Angelo E (2015). Action potential processing in a detailed Purkinje cell model reveals a critical role for axonal compartmentalization. Front. Cell Neurosci..

[34] Masoli, S. *et al.* Single neuron optimization as a basis for accurate biophysical modeling: The case of cerebellar granule cells. *Front. Cell. Neurosci.* **11**, 10.3389/fncel.2017.00071 (2017). 10.3389/fncel.2017.00071 PMC5350144 28360841

[35] Masoli S, Tognolina M, Laforenza U, Moccia F, D'Angelo E (2020). Parameter tuning differentiates granule cell subtypes enriching transmission properties at the cerebellum input stage. Commun. Biol..

[36] Lennon W, Hecht-Nielsen R, Yamazaki T (2014). A spiking network model of cerebellar Purkinje cells and molecular layer interneurons exhibiting irregular firing. Front. Comput. Neurosci..

[37] Lennon W, Yamazaki T, Hecht-Nielsen R (2015). A model of in vitro plasticity at the parallel fiber-molecular layer interneuron synapses. Front. Comput. Neurosci..

[38] De Zeeuw C (2011). Spatiotemporal firing patterns in the cerebellum. Nat. Rev. Neurosci..

[39] Arlt C, Häusser M (2020). Microcircuit rules governing impact of single interneurons on purkinje cell output in vivo. Cell Rep..

[40] Druckmann S (2007). A novel multiple objective optimization framework for constraining conductance-based neuron models by experimental data. Front. Neurosci..

[41] Dean P, Porrill J (2010). The cerebellum as an adaptive filter: A general model?. Funct. Neurol..

[42] Alcami P, Marty A (2013). Estimating functional connectivity in an electrically coupled interneuron network. Proc. Natl. Acad. Sci. USA.

[43] Hines M, Carnevale N (2001). Neuron: A tool for neuroscientists. Neuroscientist.

[44] Nieus, T. *et al.* LTP regulates burst initiation and frequency at mossy fiber-granule cell synapses of rat cerebellum: experimental observations and theoretical predictions. *J. Neurophysiol.* **95**, 686–699, 10.1152/jn.00696.2005 (2006) 10.1152/jn.00696.2005 16207782

[45] Van Geit W (2016). BluePyOpt: Leveraging open source software and cloud infrastructure to optimise model parameters in neuroscience. Front. Neuroinform..

[46] Lu H, Esquivel AV, Bower JM (2009). 3D electron microscopic reconstruction of segments of rat cerebellar Purkinje cell dendrites receiving ascending and parallel fiber granule cell synaptic inputs. J. Comp. Neurol..

[47] He Q (2015). Interneuron- and GABA(A) receptor-specific inhibitory synaptic plasticity in cerebellar Purkinje cells. Nat. Commun..

[48] Bidoret C, Bouvier G, Ayon A, Szapiro G, Casado M (2015). Properties and molecular identity of NMDA receptors at synaptic and non-synaptic inputs in cerebellar molecular layer interneurons. Front. Synaptic Neurosci..

[49] Carter A, Regehr W (2000). Prolonged synaptic currents and glutamate spillover at the parallel fiber to stellate cell synapse. J. Neurosci..

[50] Tsodyks M, Pawelzik K, Markram H (1998). Neural networks with dynamic synapses. Neural Comput..

[51] Santucci D, Raghavachari S (2008). The effects of NR2 subunit-dependent NMDA receptor kinetics on synaptic transmission and CaMKII activation. PLoS Comput. Biol..

[52] Nieus TR, Mapelli L, D'Angelo E (2014). Regulation of output spike patterns by phasic inhibition in cerebellar granule cells. Front Cell Neurosci.

[53] Zitzler E, Kunzli S (2004). Parallel Problem Solving from Nature—PPSN VIII | SpringerLink.

[54] Van Geit, W. *Blue Brain Project. eFEL*. https://github.com/BlueBrain/eFEL. (2015).

[55] Gutfreund Y, Yarom Y, Segev I (1995). Subthreshold oscillations and resonant frequency in guinea-pig cortical neurons: Physiology and modelling. J. Physiol..

[56] Rakic P (1972). Extrinsic cytological determinants of basket and stellate cell dendritic pattern in the cerebellar molecular layer. J. Comp. Neurol..

[57] Uylings H, van Eden C, Hofman M (1986). Morphometry of size/volume variables and comparison of their bivariate relations in the nervous system under different conditions. J. Neurosci. Methods.

[58] Bok S (1959). Histonomy of the Cerebral Cortex.

[59] Jacobs B (2014). Comparative neuronal morphology of the cerebellar cortex in afrotherians, carnivores, cetartiodactyls, and primates. Front Neuroanat.

[60] Bao J, Reim K, Sakaba T (2010). Target-dependent feedforward inhibition mediated by short-term synaptic plasticity in the cerebellum. J. Neurosci..

[61] Dorgans K (2019). Short-term plasticity at cerebellar granule cell to molecular layer interneuron synapses expands information processing. eLife.

[62] Grangeray-Vilmint A, Valera A, Kumar A, Isope P (2018). Short-term plasticity combines with excitation-inhibition balance to expand cerebellar purkinje cell dynamic range. J. Neurosci..

[63] Bower JM, Woolston DC (1983). Congruence of spatial organization of tactile projections to granule cell and Purkinje cell layers of cerebellar hemispheres of the albino rat: Vertical organization of cerebellar cortex. J. Neurophysiol..

[64] Nahir B, Jahr CE (2013). Activation of extrasynaptic NMDARs at individual parallel fiber-molecular layer interneuron synapses in cerebellum. J. Neurosci..

[65] Pouzat C, Marty A (1999). Somatic recording of GABAergic autoreceptor current in cerebellar stellate and basket cells. J. Neurosci..

[66] Kondo S, Marty A (1998). Synaptic currents at individual connections among stellate cells in rat cerebellar slices. J. Physiol..

[67] Herzfeld DJ, Kojima Y, Soetedjo R, Shadmehr R (2015). Encoding of action by the Purkinje cells of the cerebellum. Nature.

[68] 68Koekkoek, S. *et al.* Cerebellar LTD and learning-dependent timing of conditioned eyelid responses. S*cience (New York, N.Y.)* 3**01,**10.1126/science.1088383 (2003).

[69] D'Angelo E, De Filippi G, Rossi P, Taglietti V (1995). Synaptic excitation of individual rat cerebellar granule cells in situ: Evidence for the role of NMDA receptors. J Physiol.

[70] Forti L, Cesana E, Mapelli J, D'Angelo E (2006). Ionic mechanisms of autorhythmic firing in rat cerebellar Golgi cells. J Physiol.

[71] Dykstra S, Engbers JDT, Bartoletti TM, Turner RW (2016). J Physiol.

[72] Moscato L (2019). Long-lasting response changes in deep cerebellar nuclei in vivo correlate with low-frequency oscillations. Front Cell Neurosci.

[73] D'Angelo E (2011). The cerebellar network: from structure to function and dynamics. Brain Res Rev.

[74] Braitenberg V, Heck D, Sultan F (1997). The detection and generation of sequences as a key to cerebellar function: Experiments and theory. Behav. Brain Sci..

[75] Kim T (2016). Long-term optical access to an estimated one million neurons in the live mouse cortex. Cell Rep..

[76] Casali S, Marenzi E, Medini C, Casellato C, D'Angelo E (2019). Reconstruction and simulation of a Scaffold model of the cerebellar network. Front. Neuroinform..

[77] Zhang B (2015). Neuroligins sculpt cerebellar Purkinje-cell circuits by differential control of distinct classes of synapses. Neuron.

[78] Gall D (2005). Intracellular calcium regulation by burst discharge determines bidirectional long-term synaptic plasticity at the cerebellum input stage. J. Neurosci..

[79] D'Errico A, Prestori F, D'Angelo E (2009). Differential induction of bidirectional long-term changes in neurotransmitter release by frequency-coded patterns at the cerebellar input. J Physiol.

[80] D'Angelo E, De Zeeuw CI (2009). Timing and plasticity in the cerebellum: Focus on the granular layer. Trends Neurosci..

[81] Ito M, Kano M (1982). Long-lasting depression of parallel fiber-Purkinje cell transmission induced by conjunctive stimulation of parallel fibers and climbing fibers in the cerebellar cortex. Neurosci Lett.

[82] Lev-Ram V, Wong ST, Storm DR, Tsien RY (2002). A new form of cerebellar long-term potentiation is postsynaptic and depends on nitric oxide but not cAMP. Proc Natl Acad Sci U S A.

[83] Salin P, Malenka R, Nicoll R (1996). Cyclic AMP mediates a presynaptic form of LTP at cerebellar parallel fiber synapses. Neuron.

[84] Coesmans M, Weber JT, De Zeeuw CI, Hansel C (2004). Bidirectional parallel fiber plasticity in the cerebellum under climbing fiber control. Neuron.

[85] Qiu DL, Knöpfel T (2009). Presynaptically expressed long-term depression at cerebellar parallel fiber synapses. Pflugers Arch.

[86] Liu SJ, Lachamp P, Liu Y, Savtchouk I, Sun L (2008). Long-term synaptic plasticity in cerebellar stellate cells. Cerebellum.

[87] Rancillac A, Crépel F (2004). Synapses between parallel fibres and stellate cells express long-term changes in synaptic efficacy in rat cerebellum. J Physiol.

[88] Bender VA, Pugh JR, Jahr CE (2009). Presynaptically expressed long-term potentiation increases multivesicular release at parallel fiber synapses. J Neurosci.

[89] Kawaguchi SY, Hirano T (2007). Sustained structural change of GABA(A) receptor-associated protein underlies long-term potentiation at inhibitory synapses on a cerebellar Purkinje neuron. J Neurosci.

[90] Kano M, Rexhausen U, Dreessen J, Konnerth A (1992). Synaptic excitation produces a long-lasting rebound potentiation of inhibitory synaptic signals in cerebellar Purkinje cells. Nature.

[91] Hirano T, Kawaguchi S (2014). Regulation and functional roles of rebound potentiation at cerebellar stellate cell-Purkinje cell synapses. Front. Cell. Neurosci..

[92] Mapelli L, Pagani M, Garrido JA, D'Angelo E (2015). Integrated plasticity at inhibitory and excitatory synapses in the cerebellar circuit. Front Cell Neurosci.

[93] Gao Z, van Beugen BJ, De Zeeuw CI (2012). Distributed synergistic plasticity and cerebellar learning. Nat Rev Neurosci.

[94] D'Angelo E (2014). The organization of plasticity in the cerebellar cortex: from synapses to control. Prog Brain Res.

[95] Casali, S., Tognolina, M. & D’Angelo, E. Cellular-resolution mapping uncovers spatial adaptive filtering at the cerebellum input stage. B*ioRxiv*. 10.1101/2020.03.14.991794 (2020). 10.1038/s42003-020-01360-y PMC7599228 33128000

[96] Diwakar S, Lombardo P, Solinas S, Naldi G, D'Angelo E (2011). Local field potential modeling predicts dense activation in cerebellar granule cells clusters under LTP and LTD control. PLoS ONE.

[97] Bower JM (2002). The organization of cerebellar cortical circuitry revisited: implications for function. Ann N Y Acad Sci.

[98] Ros H, Sachdev RN, Yu Y, Sestan N, McCormick DA (2009). Neocortical networks entrain neuronal circuits in cerebellar cortex. J Neurosci.

[99] Courtemanche R, Robinson JC, Aponte DI (2013). Linking oscillations in cerebellar circuits. Front Neural Circuits.

[100] Casali S, Marenzi E, Medini C, Casellato C (2019). Reconstruction and simulation of a Scaffold model of the cerebellar network. Front. Neuroinform..

