# Supplementary material for: Stellate cell computational modeling predicts signal filtering in the molecular layer circuit of cerebellum
Source: Sci Rep. 2021 Feb 16;11:3873. doi: 10.1038/s41598-021-83209-w (PMC7886897; doi:10.1038/s41598-021-83209-w)
Supplement: Supplementary file 5 — Supplementary Information. [file 41598_2021_83209_MOESM5_ESM.pdf]

SUPPLEMENTARY INFORMATION

Stellate cell computational modeling predicts signal filtering in the molecular layer circuit of cerebellum

Martina Francesca Rizza[1], Francesca Locatelli[1], Stefano Masoli[1], Diana Sánchez-Ponce[3], Alberto Muñoz[3,4], Francesca Prestori[1†], Egidio D'Angelo[1,2] *†

[1] Department of Brain and Behavioral Sciences, University of Pavia, Italy

[2] Brain Connectivity Center, IRCCS Mondino Foundation, Pavia, Italy

[3] Centro de Tecnología Biomédica (CTB), Universidad Politécnica de Madrid, Spain

[4] Departamento de Biología Celular, Universidad Complutense, Madrid, Spain

[†] co-last authors

List of abbreviations used below:

- SC, stellate cell
- PC, Purkinje cell
- AIS, axon initial segment
- Dendprox, proximal dendrites
- Denddist, distal dendrites
- AP, action potential
- AHP, afterhyperpolarization
- Thr, threshold
- Ampl, amplitude
- HW, half-width
- Freq, frequency
- eFEL, Electrophys Feature Extraction Library

Ionic channels

Nav1.1 - Nav1.6. Expression and distribution of SC sodium channels were determined experimentally [1,2]. Nav1.1 channel was placed on the soma and Nav1.6 on the AIS and axonal compartment. The gating mechanism was taken from [3,4].

Kv1.1. The low threshold Kv1.1 channel, in accordance with experiments [1,5,6], was placed on all compartments. The gating mechanism was taken from [7].

Kv3.4. This ionic channel with delayed rectifier properties was distributed on the soma, AIS and axon to repolarize the Na^+ spikes [8-10]. The gating mechanism was taken from [7].

Kv4.3. This ionic channel with A-type properties was placed on the soma and proximal dendrites [11-13]. Moreover, Kv4.3 interacts with the LVA Ca^{2+} (Cav3.x) channels to create a complex with important functions in SC firing [14]. The gating mechanism was taken from [15].

Kv7.x. The M-current was identified electrophysiologically [16-18]. Kv7 channels were expressed in the SC AIS were placed in the AIS using gating mechanisms developed for the granule cell [19].

Kir. Inward rectifier K^+ channel was expressed in the SC soma [20-22].

KCa1.1 - KCa2.2. Large and small conductance calcium-activated potassium channels, which can cluster with Cav2.1 channels, were placed on the proximal/distal dendritic and somatic compartments based on immunohistochemical and electrophysiological data [14,23-25]. The gating mechanism was taken from [26].

Cav2.1. The high-threshold calcium channels (P-type) were placed on the proximal/distal dendritic and somatic compartments [27,28]. The gating mechanism of Cav2.1 was taken from [26,29].

Cav3.2 – Cav3.3. The low-threshold calcium channels (T-type) were placed on the proximal dendritic and somatic compartments [11,12,14,30,31]. The gating mechanism of Cav2.1 was taken from [32,33].

HCN1. Hyperpolarization activated cyclic nucleotide-gated cationic channel) was placed on the AIS, somatic and axonal compartments [34,35]. The gating mechanism was taken from [36,37].

Calcium dynamics. The calcium buffer was taken from [15] and modified to contain Parvalbumin, the typical calcium binding proteins of the SC [26,38-40].

SUPPLEMENTARY TABLES

Supplementary Table 1. Ionic mechanisms in stellate cell models

Ionic Channel	Location	Range Gi-max (mS/cm^2)	E_{rev} (mV)
Nav1.1	Soma	$1e^{-1}$ - $4e^{-1}$	60
Nav1.6	Ais<br>Axon	$6e^{-1}$ - $8e^{-1}$<br>$6e^{-3}$ -$9e^{-3}$	60
Kv3.4	Soma<br>AIS<br>Axon	$5e^{-3}$ - $8e^{-3}$<br>$2e^{-2}$ - $4e^{-2}$<br>$1e^{-2}$ - $3e^{-2}$	-84
Kv4.3	Soma<br>Dendprox	$4e^{-3}$ - $6e^{-3}$<br>$1e^{-3}$ - $4e^{-3}$	-84
Kv1.1	Soma<br>AIS<br>Axon<br>Dendprox<br>Denddist	$6e^{-4}$ - $4e^{-3}$<br>$3e^{-3}$ - $5e^{-3}$<br>$2.5e^{-3}$ - $5e^{-3}$<br>$4e^{-3}$ - $1e^{-2}$<br>$1e^{-3}$ - $3e^{-3}$	-84
Kir2.3	Soma	1e-5 - 5e-5	-84
Kv7	AIS	6e-5 - 8e-5	-84
KCa1.1	Soma<br>Dendprox<br>Denddist	$4e^{-3}$ - $9e^{-3}$<br>$1e^{-3}$ - $5e^{-3}$<br>$1e^{-3}$ - $4e^{-3}$	-84
KCa2.2	Soma<br>Dendprox<br>Denddist	4e-4 - 9e-4<br>3e-06 - 4.5e-06<br>1e-05 - 2e-05	-84
Cav2.1	Soma<br>Dendprox<br>Denddist	$2e^{-4}$ - $3.5e^{-4}$<br>$4e^{-4}$ - $7e^{-4}$<br>$2e^{-}4$ - $4e^{-4}$	137.5
Cav3.2	Soma<br>Dendprox	$9e^{-4}$ - $2e^{-3}$<br>$7e^{-4}$ - $1e^{-3}$	137.5
Cav3.3	Soma<br>Dendprox	$1.5e^{-05}$ - $2e^{-05}$<br>$1e^{-05}$ - $2e^{-05}$	137.5
HCN1	Soma<br>AIS<br>Axon	$2e^{-4}$ - $7e^{-4}$<br>$7e^{-4}$ - $1e^{-3}$<br>$6e^{-4}$ - $1e^{-3}$	-34
Leak	Soma<br>AIS<br>Axon<br>Dendprox<br>Denddist	3e-5	-48

The table shows the main properties of ionic channels used in the SC models. For each ionic channel type, the columns specify the maximum ionic conductance (G_i-max), ionic channels reversal potential (E_{rev}). The corresponding gating equations were written either in Hodgkin-Huxley (HH) style or in Markovian style.

Supplementary Table 2. Electrotonic compartments in stellate cell models

Compartment	1 stellate cell	2 stellate cell	3 stellate cell	4 stellate cell
Soma	1 section; area 37.3 μm^2	1 section; area 31.1 μm^2	1 section; area 70.1 μm^2	1 section; area 31.1 μm^2
Proximal dendrites	14 sections	7 sections	5 sections	18 sections
Distal dendrites	90 sections	35 sections	85 sections	44 sections
Total length dendrites	1162.8 μm	755.1 μm	875.7 μm	587.4 μm
AIS	1 section; length 25.5 μm	1 section; length 13.8 μm	1 section; length 46.6 μm	1 section; length 26.6 μm
Axon	14 sections; Length 191.0 μm	20 sections; Length 361.1 μm	76 sections; Length 1218.0 μm	31 sections; Length 551.4 μm

The table shows the morphological analysis with NEURON software of the four morphologies used for the multi-compartment SC models. The table reports the sections of the multi-compartment SC model along with their number, their length and the soma area.

Supplementary Table 3. Synaptic model parameters

	SC	SC	SC	PC
	AMPA receptor	NMDA receptor	$GABA_A$ receptor	AMPA receptor
Gmax (mS/cm^2)	2300	10000	1600	1200
P	0.15	0.15	0.42	0.13
τ facil (ms)	10.8	5	4	54
τ recov (ms)	35.1	8	38.7	35.1

The table summarizes the parameters used for modeling the AMPA, NMDA and $GABA_A$ receptors [41-43].

Supplementary Table 4, 5. Spike features

Spontaneous firing

	Clampfit EXP (n=9)	eFEL EXP (n=9)	eFEL MOD (n=4)
AP_{ampl} (mV) p=0.04	42.6 ± 3.6	37.3 ± 5.2	59.1 ± 6.3*
AP_{AHP} (mV) p=0.24	-50.4 ± 2.1	-44.2 ± 4.0	-42.7 ± 0.8
AP_{Thr} (mV) p=0.29	-34.2 ± 1.1	-31.8 ± 1.3	-31.8 ± 0.6
AP_{HW} (ms) p=0.26	0.76 ± 0.05	1.0 ± 0.2	1.1 ± 0.04
AP Freq (Hz) p=0.84	24.2 ± 2.1	25.1 ± 2.1	22.8 ± 3.3
V_m (mV) p=0.95	-41.7 ± 1.8	-41.2 ± 1.2	-40.9 ± 0.8

Injected current (16 pA)

	Clampfit EXP (n=5)	eFEL EXP (n=5)	eFEL MOD (n=4)
AP_{Ampl} (mV) p=0.01	33.7 ± 4.8	32.8 ± 5.0	57.7 ± 5.7**
AP_{AHP} (mV) p=0.55	-46.4 ± 5.2	-46.6 ± 5.6	-39.6 ± 0.6
AP_{Thr} (mV) p=0.97	-31.3 ± 2.1	-30.3 ± 5.1	-30.3 ± 0.6
AP_{HW} (ms) p=0.4	0.96 ± 0.16	0.98 ± 0.14	1.2 ± 0.1
AP Freq (Hz) p=0.06	80.4± 6.9	79.4 ± 7.6	54.4 ± 6.3

The tables show exemplar values of features, obtained from experimental traces (n = 9 used for the spontaneous firing recordings and n = 5 used for the current injection experimental protocols) and from simulations (n = 4) using eFEL and Clampfit software.

SUPPLEMENTARY FIGURES

Supplementary Figure 1. Ionic currents in stellate cell model sections

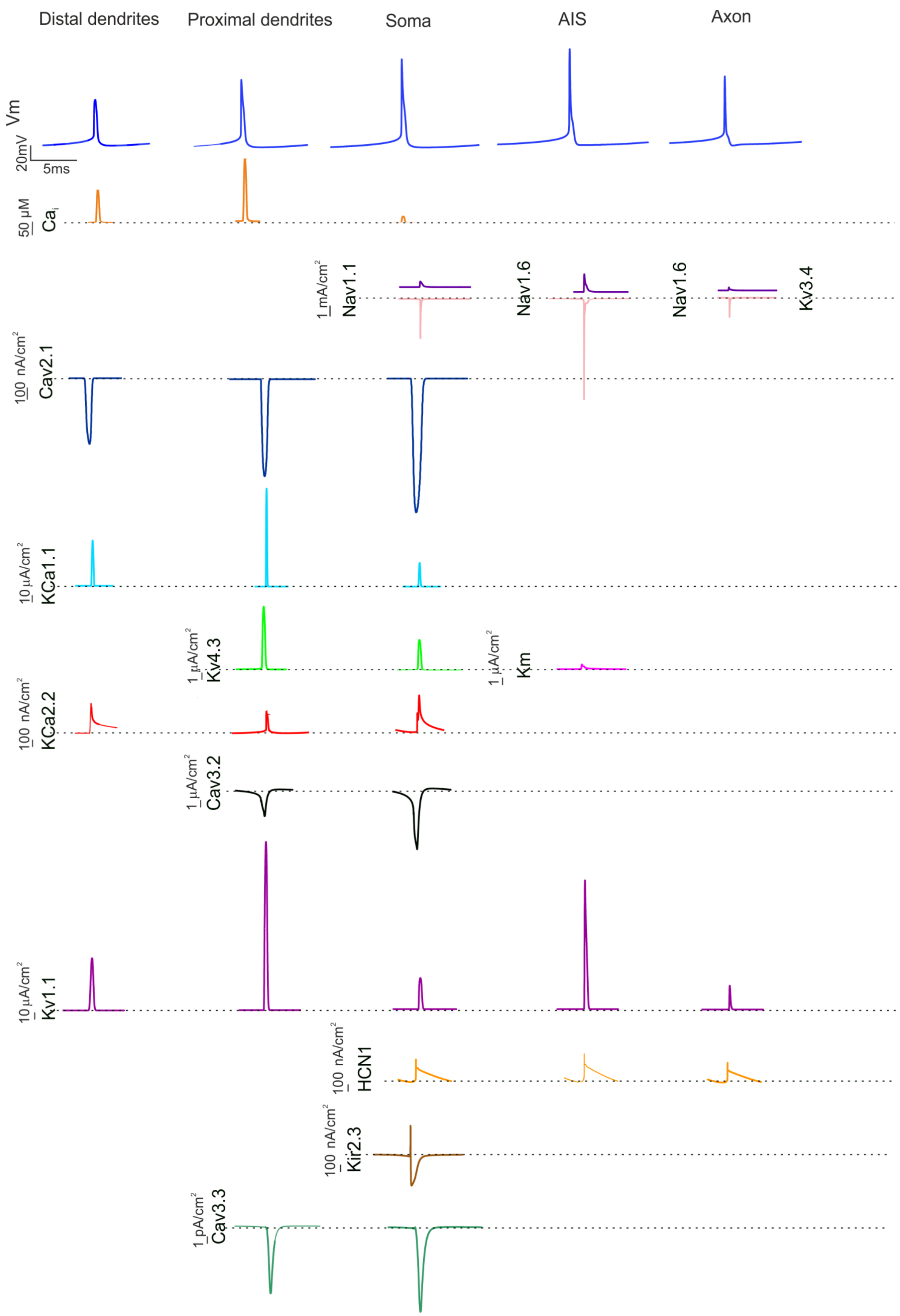

The traces show the ionic currents and calcium concentration changes generated by membrane channels in the SC model when a spike occurs during autorhythmic firing. Note the localization of channels in different sections and the different calibration scales.

Supplementary Figure 2. Ionic currents in the somatic compartment in response to current injection

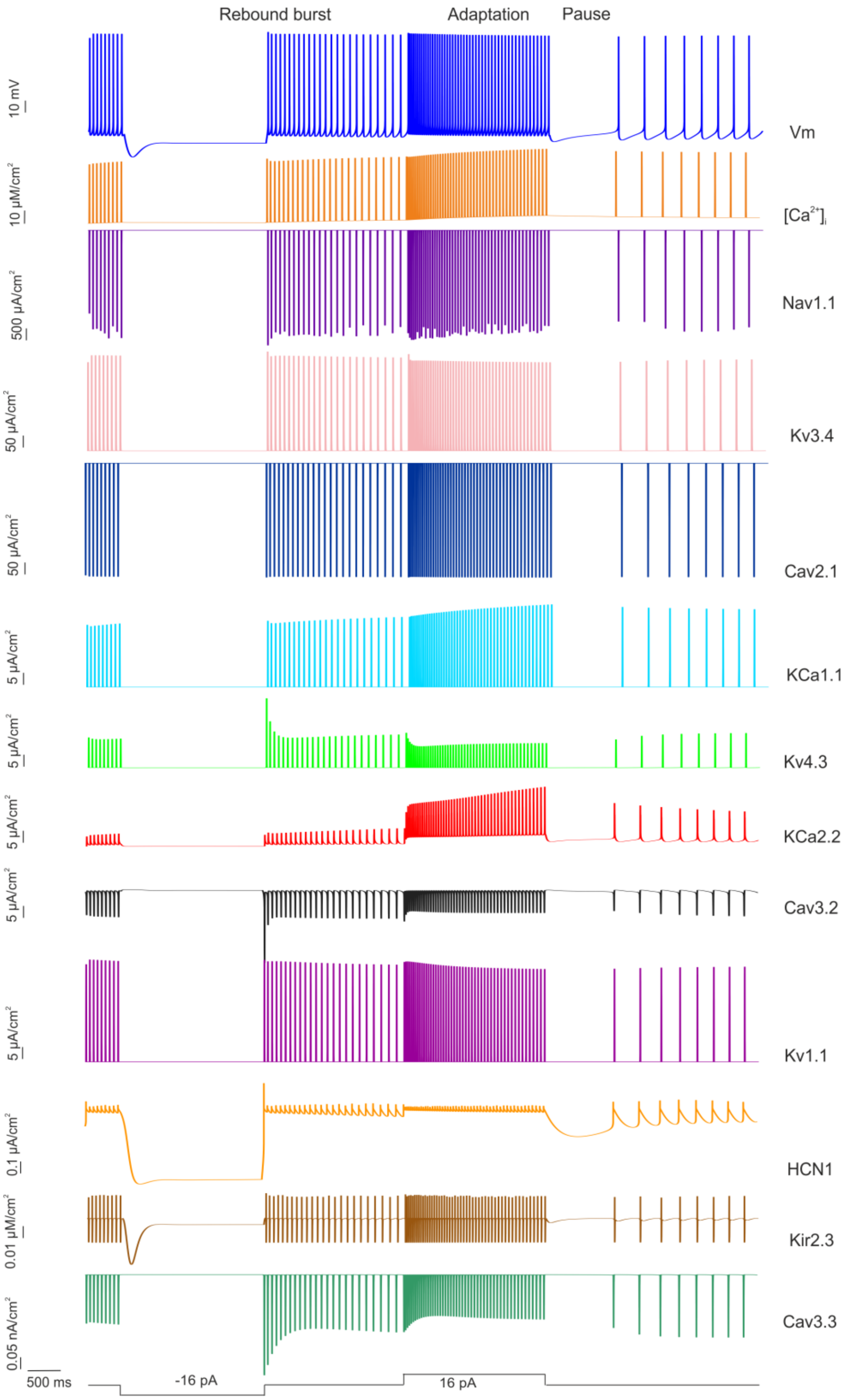

The traces show the model response recorded from the soma during alternated phases of pacemaking, hyperpolarization and depolarization. The upper traces shows membrane potential (V_m) and $[Ca^{2+}]_i$, the other traces show the ionic currents. It should be noted that marked changes in current size are correlated with rebound bursts, adaptation and pauses.

Supplementary Figure 3. AMPA-NMDA-GABA$_A$ receptors

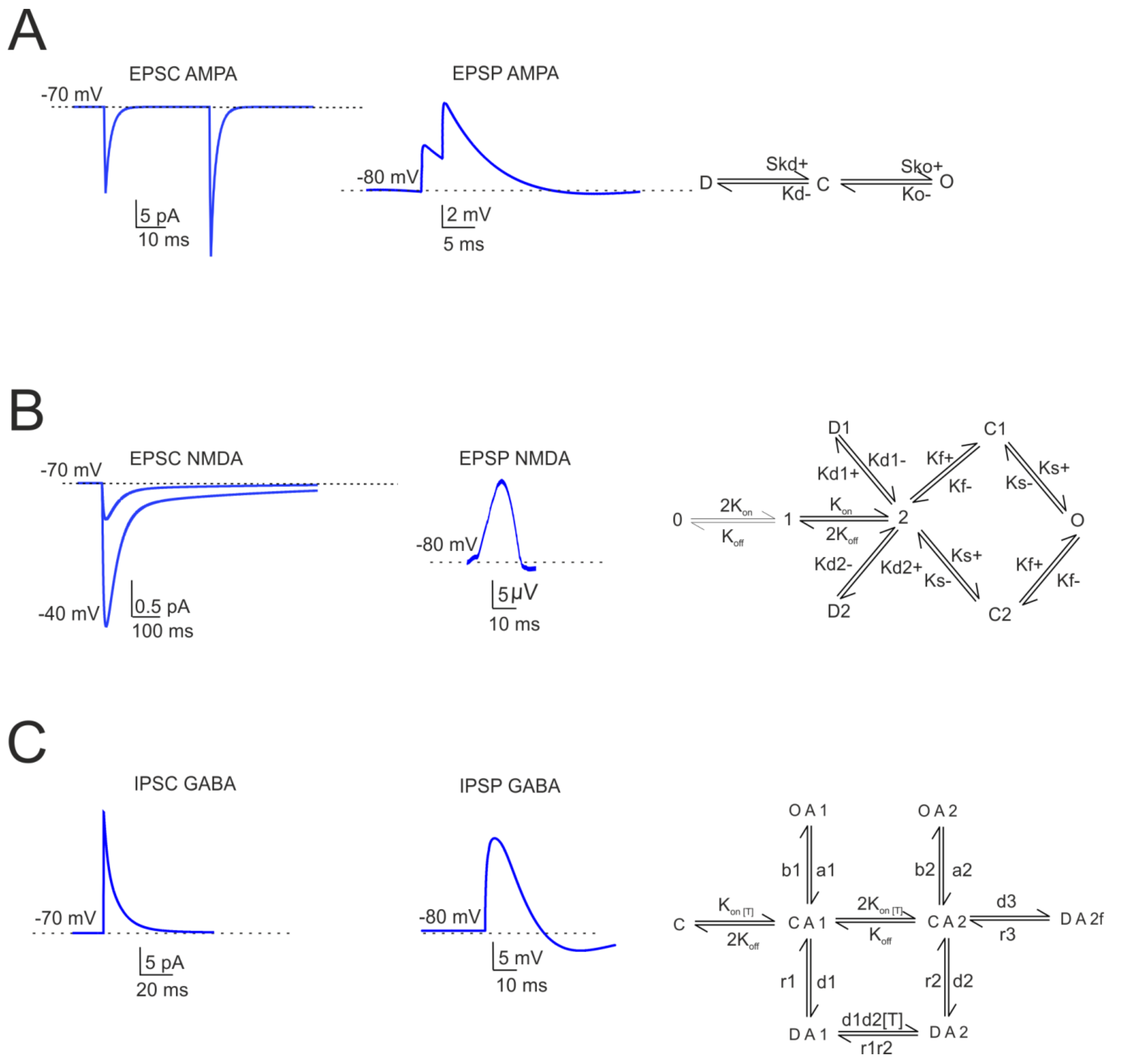

(A) The traces show simulated AMPA EPSC and EPSP. The AMPA receptor-channels kinetic scheme is shown on the left. (B) The traces show simulated NMDA EPSC and EPSP. The NMDA receptor-channels kinetic scheme is shown on the left. (C) The traces show simulated GABA-A IPSC and IPSP. The GABA-A receptor-channels kinetic scheme is shown on the left.

Supplementary Figure 4. Dendritic currents in response to current injection

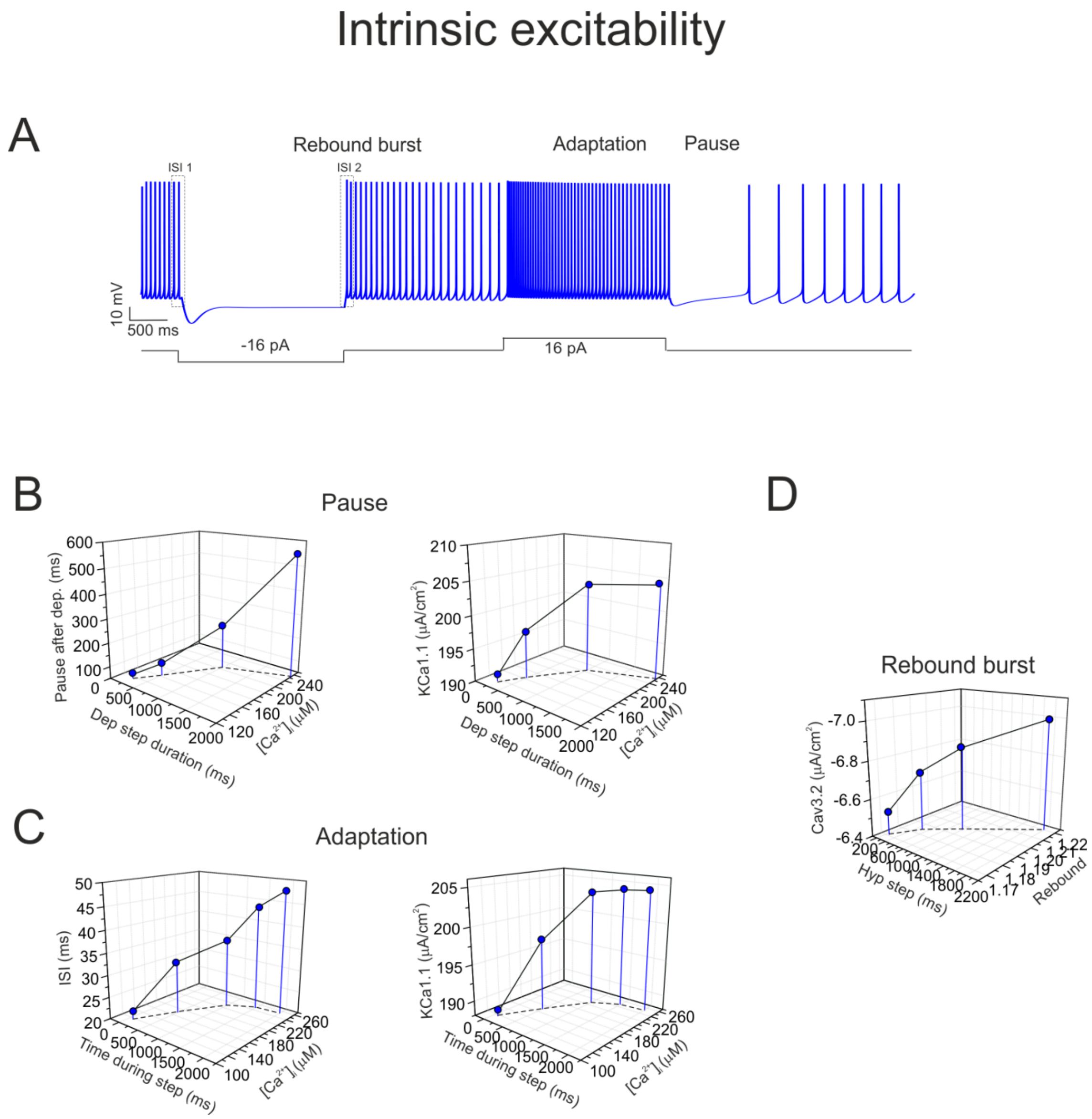

The figure shows the main dendritic mechanisms correlated with injected current pulses of different duration.

(A) The trace shows the model response during alternated phases of pacemaking, hyperpolarization and depolarization (as in Supplementary Fig. 2).

(B) The 3D plots show that the pause increases with the duration of the depolarizing step, the increase in $[Ca^{2+}]_i$ and the size of the KCa1.1 current.

(C) The 3D plots show that adaptation increases during the 2000ms-depolarizing step along with $[Ca^{2+}]_i$ and KCa1.1 current.

(D) The 3D plot shows that the rebound burst (ISI2/ISI1) increases with the duration of the hyperpolarizing step and the size of the Cav3.2 current.

__Supplementary Figure 5. Dendritic currents in response to repetitive synaptic transmission__

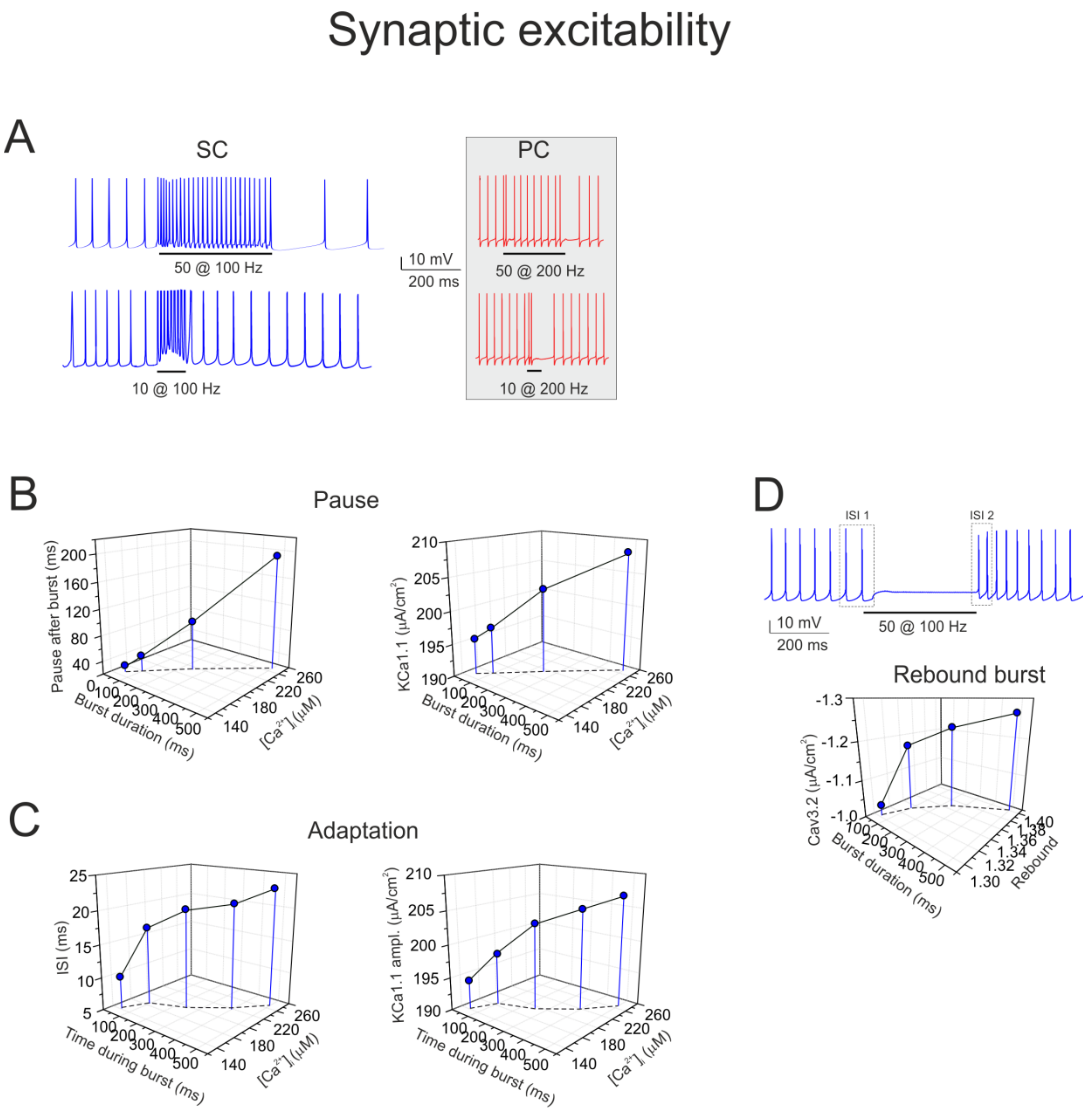

The figure shows the main dendritic mechanisms correlated with bursts of synaptic activity.

(A) After a short burst (10 pulses @ 100 Hz; the trace is replotted from Fig. 6A), the SC model does not make any pause. After a long duration burst (50 pulses @ 100 Hz), the SC model shows a pause. These properties resemble those appearing at the end of a prolonged depolarizing current injection of the same duration (cf. Fig. 3 and Supplementary Fig. 4). For comparison, the figure compares the PC model, which shows a pause following bursts of both short and long duration demonstrating that the behavior of stellate cells reflects the specific balance, composition and localization of their ionic channels.

(B) The 3D plots show that the pause increases with burst duration (@100Hz , 3 synapses PF→SC), the increase in $[Ca^{2+}]_i$ and the size of the KCa1.1 current.

(C) The 3D plots show that the adaptation increases during the 500ms-burst (@100Hz , 3 synapses PF→SC), along with $[Ca^{2+}]_i$ and KCa1.1 current.

(D) Top, the trace shows the SC model response during inhibitory burst duration (@100Hz , 32 synapses SC→ SC). Bottom, the 3D plots show rebound burst (ISI2/ISI1) increases with the inhibitory burst duration (@100Hz , 32 synapses SC→SC) and the size of the Cav3.2 current.

Supplementary Figure 6. Simulation of long-duration EPSC trains in SCs

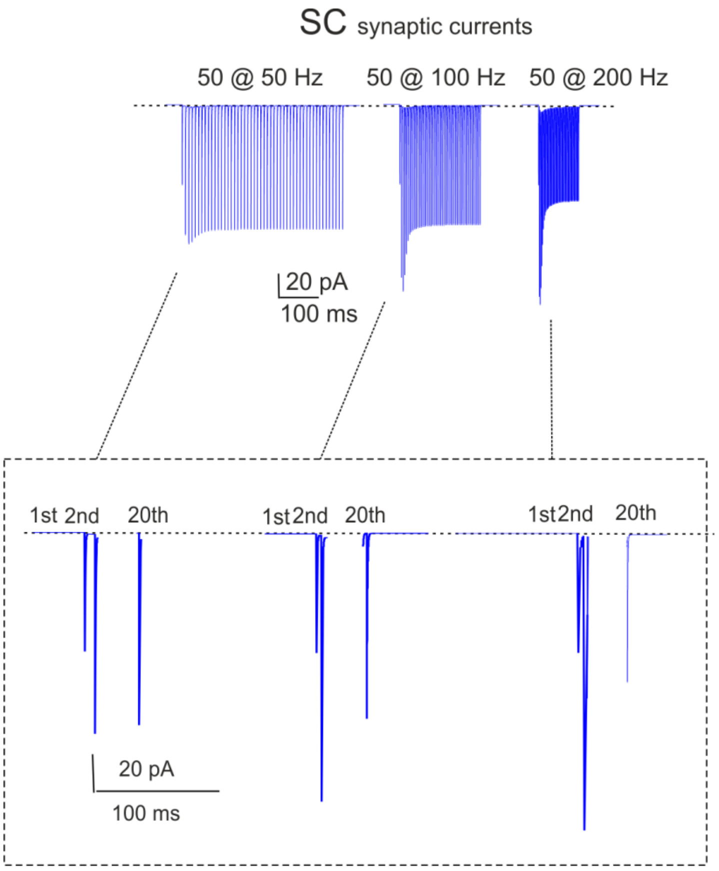

Simulated synaptic currents in a SC evoked by activation of 3 PF-SC synapses with long input bursts. Note that, at all tested frequencies, the spike amplitude decreases after the initial increase but remains over the control level.

Supplementary Figure 7.

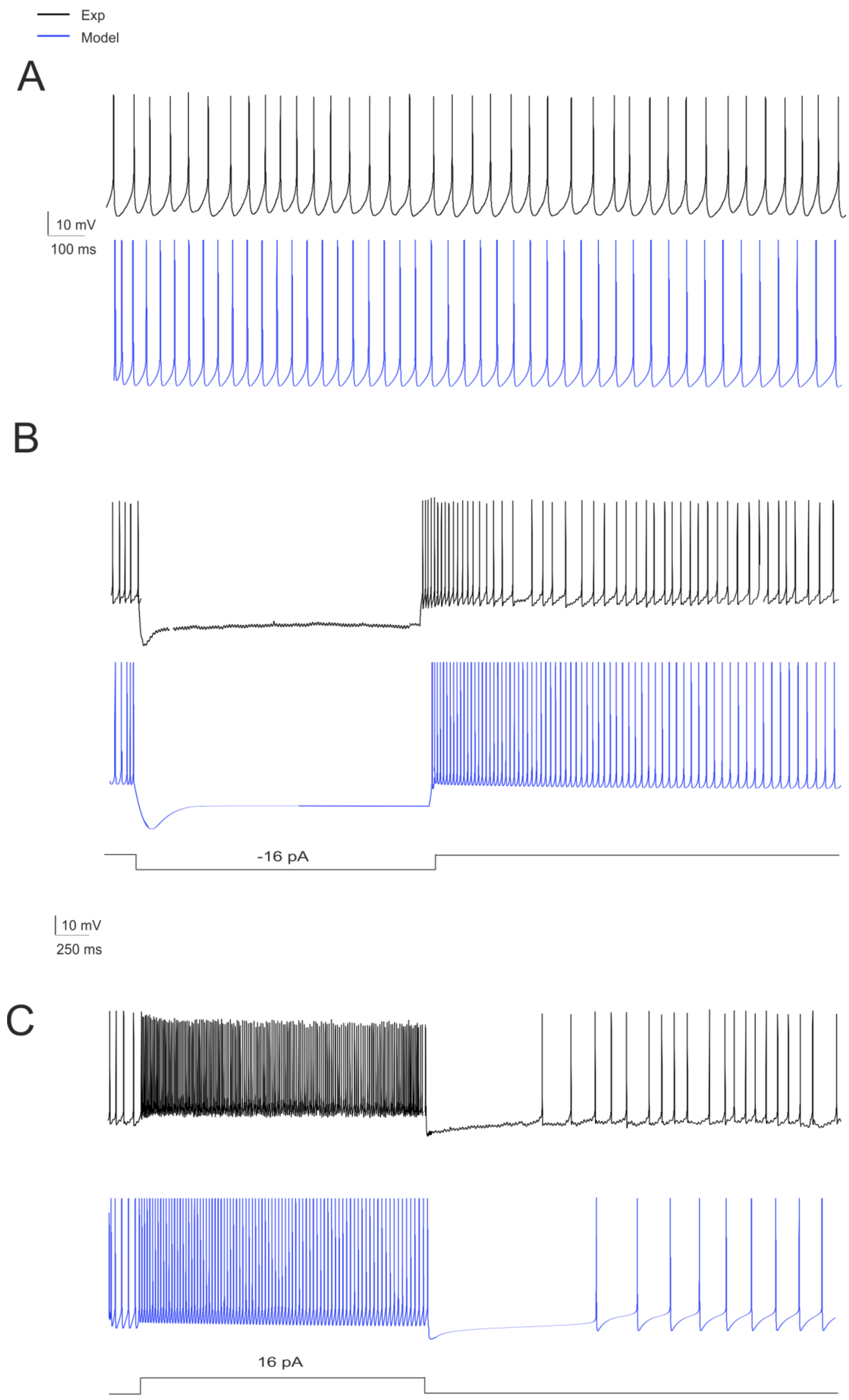

Enlargement of voltage traces showed in Fig. 2A, 3A and 3C.

Supplementary Movie 1. Stellate cell morphology

The movie shows a SC morpho-electrical equivalent (morphology 1 in Fig. 1). The dendritic tree was flatted on the sagittal plane of the *folium* and the axon, after an initial part travelling parallel to the dendrite, advanced along the transverse plane.

Supplementary Movie 2. Stellate cell pacemaker activity

The movie shows the SC model spontaneous activity (membrane potential in the soma).

Supplementary Movie 3. Parallel fibers – stellate cell – Purkinje cell activity

SC model activation by a PF burst (10 impulses @ 200 Hz). The PC receives SC inhibition and generates a pause. The plots show membrane potential traces taken in the SC and PC soma.

Supplementary Movie 4. Parallel fibers – stellate cell – Purkinje cell activity

SC and PC models activation by a PF burst (10 impulses @ 200 Hz). The PC model receives both SC inhibition and PF excitation and generates a burst-pause. The plots show membrane potential traces taken in the SC and PC soma.

SUPPLEMENTARY REFERENCES

1 Lorincz, A. & Nusser, Z. Cell-type-dependent molecular composition of the axon initial segment. *The Journal of neuroscience : the official journal of the Society for Neuroscience* **28**, doi:10.1523/JNEUROSCI.4833-08.2008 (2008).

2 Schaller, K. & Caldwell, J. Expression and distribution of voltage-gated sodium channels in the cerebellum. *Cerebellum (London, England)* **2**, doi:10.1080/14734220309424 (2003).

3 Khaliq, Z., Gouwens, N. & Raman, I. The contribution of resurgent sodium current to high-frequency firing in Purkinje neurons: an experimental and modeling study. *The Journal of neuroscience : the official journal of the Society for Neuroscience* **23**, doi:10.1523/JNEUROSCI.23-12-04899.2003 (2003).

4 Magistretti, J., Castelli, L., Forti, L. & D'Angelo, E. Kinetic and functional analysis of transient, persistent and resurgent sodium currents in rat cerebellar granule cells in situ: an electrophysiological and modelling study. *J Physiol* **573**, 83-106, doi:10.1113/jphysiol.2006.106682 (2006).

5 Southan, A. & Robertson, B. Electrophysiological characterization of voltage-gated K(+) currents in cerebellar basket and purkinje cells: Kv1 and Kv3 channel subfamilies are present in basket cell nerve terminals. *The Journal of neuroscience : the official journal of the Society for Neuroscience* **20**, doi:10.1523/JNEUROSCI.20-01-00114.2000 (2000).

6 Williams, M., Fuchs, J., Green, J. & Morielli, A. Cellular mechanisms and behavioral consequences of Kv1.2 regulation in the rat cerebellum. *The Journal of neuroscience : the official journal of the Society for Neuroscience* **32**, doi:10.1523/JNEUROSCI.6504-11.2012 (2012).

7 Akemann, W. & Knöpfel, T. Interaction of Kv3 potassium channels and resurgent sodium current influences the rate of spontaneous firing of Purkinje neurons. *The Journal of neuroscience : the official journal of the Society for Neuroscience* **26**, doi:10.1523/JNEUROSCI.5204-05.2006 (2006).

8 Rowan, M., Tranquil, E. & Christie, J. Distinct Kv channel subtypes contribute to differences in spike signaling properties in the axon initial segment and presynaptic boutons of cerebellar interneurons. *The Journal of neuroscience : the official journal of the Society for Neuroscience* **34**, doi:10.1523/JNEUROSCI.4208-13.2014 (2014).
9 Brooke, R., Atkinson, L., Edwards, I., Parson, S. & Deuchars, J. Immunohistochemical localisation of the voltage gated potassium ion channel subunit Kv3.3 in the rat medulla oblongata and thoracic spinal cord. *Brain research* **1070**, doi:10.1016/j.brainres.2005.10.102 (2006).
10 Perney, T., Marshall, J., Martin, K., Hockfield, S. & Kaczmarek, L. Expression of the mRNAs for the Kv3.1 potassium channel gene in the adult and developing rat brain. *Journal of neurophysiology* **68**, doi:10.1152/jn.1992.68.3.756 (1992).
11 Molineux, M. L., Fernandez, F. R., Mehaffey, W. H. & Turner, R. W. A-type and T-type currents interact to produce a novel spike latency-voltage relationship in cerebellar stellate cells. *J Neurosci* **25**, 10863-10873, doi:10.1523/jneurosci.3436-05.2005 (2005).
12 Anderson, D. *et al.* The Cav3-Kv4 complex acts as a calcium sensor to maintain inhibitory charge transfer during extracellular calcium fluctuations. *J Neurosci* **33**, 7811-7824, doi:10.1523/jneurosci.5384-12.2013 (2013).
13 Alexander, R. P. D., Mitry, J., Sareen, V., Khadra, A. & Bowie, D. Cerebellar Stellate Cell Excitability Is Coordinated by Shifts in the Gating Behavior of Voltage-Gated Na(+) and A-Type K(+) Channels. *eNeuro* **6**, doi:10.1523/eneuro.0126-19.2019 (2019).
14 Turner, R. & Zamponi, G. T-type channels buddy up. *Pflugers Archiv : European journal of physiology* **466**, doi:10.1007/s00424-013-1434-6 (2014).
15 Masoli, S., Solinas, S. & D'Angelo, E. Action potential processing in a detailed Purkinje cell model reveals a critical role for axonal compartmentalization. *Front Cell Neurosci* **9**, 47, doi:10.3389/fncel.2015.00047 (2015).
16 Brown, D. & Passmore, G. Neural KCNQ (Kv7) channels. *British journal of pharmacology* **156**, doi:10.1111/j.1476-5381.2009.00111.x (2009).
17 Pan, Z. *et al.* A common ankyrin-G-based mechanism retains KCNQ and NaV channels at electrically active domains of the axon. *The Journal of neuroscience : the official journal of the Society for Neuroscience* **26**, doi:10.1523/JNEUROSCI.4314-05.2006 (2006).
18 Miceli, F., Vargas, E., Bezanilla, F. & Taglialatela, M. Gating currents from Kv7 channels carrying neuronal hyperexcitability mutations in the voltage-sensing domain. *Biophysical journal* **102**, doi:10.1016/j.bpj.2012.02.004 (2012).
19 D'Angelo, E. *et al.* Theta-frequency bursting and resonance in cerebellar granule cells: experimental evidence and modeling of a slow k+-dependent mechanism. *J Neurosci* **21**, 759-770 (2001).
20 Stonehouse, A. *et al.* Characterisation of Kir2.0 proteins in the rat cerebellum and hippocampus by polyclonal antibodies. *Histochemistry and cell biology* **112**, doi:10.1007/s004180050429 (1999).
21 Prüss, H., Derst, C., Lommel, R. & Veh, R. Differential distribution of individual subunits of strongly inwardly rectifying potassium channels (Kir2 family) in rat brain. *Brain research. Molecular brain research* **139**, doi:10.1016/j.molbrainres.2005.05.006 (2005).
22 Raphemot, R. *et al.* Discovery, Characterization, and Structure–Activity Relationships of an Inhibitor of Inward Rectifier Potassium (Kir) Channels with Preference for Kir2.3, Kir3.X, and Kir7.1. *Front Pharmacol* **2**, doi:10.3389/fphar.2011.00075 (2011).
23 Kaufmann, W., Kasugai, Y., Ferraguti, F. & Storm, J. in *Neuroscience* Vol. 169 974-986 (2010).
24 Rehak, R. *et al.* in *PLoS One* Vol. 8 (2013).
25 Womack, M. D., Hoang, C. & Khodakhah, K. Large conductance calcium-activated potassium channels affect both spontaneous firing and intracellular calcium concentration in

cerebellar Purkinje neurons. *Neuroscience* **162**, 989-1000, doi:10.1016/j.neuroscience.2009.05.016 (2009).
26 Anwar, H., Hong, S. & De Schutter, E. Controlling Ca2+-activated K+ channels with models of Ca2+ buffering in Purkinje cells. *Cerebellum (London, England)* **11**, doi:10.1007/s12311-010-0224-3 (2012).
27 Kulik, A. *et al.* Immunocytochemical localization of the alpha 1A subunit of the P/Q-type calcium channel in the rat cerebellum. *The European journal of neuroscience* **19**, doi:10.1111/j.0953-816X.2004.03319.x (2004).
28 Indriati, D. W. *et al.* in *J Neurosci* Vol. 33 3668-3678 (2013).
29 Swensen, A. M. & Bean, B. P. Ionic Mechanisms of Burst Firing in Dissociated Purkinje Neurons. *J Neurosci* **23**, 9650-9663, doi:10.1523/jneurosci.23-29-09650.2003 (2003).
30 Molineux, M. L. *et al.* Specific T-type calcium channel isoforms are associated with distinct burst phenotypes in deep cerebellar nuclear neurons. *Proc Natl Acad Sci U S A* **103**, 5555-5560, doi:10.1073/pnas.0601261103 (2006).
31 Cain, S. & Snutch, T. Contributions of T-type calcium channel isoforms to neuronal firing. *Channels (Austin, Tex.)* **4**, doi:10.4161/chan.4.6.14106 (2010).
32 Huguenard, J. & McCormick, D. Simulation of the currents involved in rhythmic oscillations in thalamic relay neurons. *Journal of neurophysiology* **68**, doi:10.1152/jn.1992.68.4.1373 (1992).
33 Xu, J. & Clancy, C. E. in *PLoS One* Vol. 3 (2008).
34 Angelo, K., London, M., Christensen, S. & Häusser, M. Local and global effects of I(h) distribution in dendrites of mammalian neurons. *The Journal of neuroscience : the official journal of the Society for Neuroscience* **27**, doi:10.1523/JNEUROSCI.5284-06.2007 (2007).
35 Luján, R., Albasanz, J., Shigemoto, R. & Juiz, J. Preferential localization of the hyperpolarization-activated cyclic nucleotide-gated cation channel subunit HCN1 in basket cell terminals of the rat cerebellum. *The European journal of neuroscience* **21**, doi:10.1111/j.1460-9568.2005.04043.x (2005).
36 Solinas, S. *et al.* Computational reconstruction of pacemaking and intrinsic electroresponsiveness in cerebellar Golgi cells. *Front Cell Neurosci* **1**, 2, doi:10.3389/neuro.03.002.2007 (2007).
37 Solinas, S. *et al.* Fast-reset of pacemaking and theta-frequency resonance patterns in cerebellar golgi cells: simulations of their impact in vivo. *Front Cell Neurosci* **1**, 4, doi:10.3389/neuro.03.004.2007 (2007).
38 Alcami, P. & Marty, A. Estimating functional connectivity in an electrically coupled interneuron network. *Proceedings of the National Academy of Sciences of the United States of America* **110**, doi:10.1073/pnas.1310983110 (2013).
39 Collin, T. *et al.* Developmental changes in parvalbumin regulate presynaptic Ca2+ signaling. *The Journal of neuroscience : the official journal of the Society for Neuroscience* **25**, doi:10.1523/JNEUROSCI.3748-04.2005 (2005).
40 Bastianelli, E. Distribution of calcium-binding proteins in the cerebellum. *Cerebellum* **2**, 242-262, doi:10.1080/14734220310022289 (2003).
41 Nieus, T. *et al.* LTP regulates burst initiation and frequency at mossy fiber-granule cell synapses of rat cerebellum: experimental observations and theoretical predictions. *J Neurophysiol* **95**, 686-699, doi:00696.2005 [pii]10.1152/jn.00696.2005 (2006).
42 Nieus, T. R., Mapelli, L. & D'Angelo, E. Regulation of output spike patterns by phasic inhibition in cerebellar granule cells. F*ront Cell Neurosci* 8**,** 246, doi:10.3389/fncel.2014.00246 (2014).
43 Santucci, D. & Raghavachari, S. The effects of NR2 subunit-dependent NMDA receptor kinetics on synaptic transmission and CaMKII activation. P*LoS computational biology* 4**,** doi:10.1371/journal.pcbi.1000208 (2008).
